# A global meta-analysis of animal manure application and soil microbial ecology based on random control treatments

**DOI:** 10.1371/journal.pone.0262139

**Published:** 2022-01-21

**Authors:** Zhenhua Guo, Lei Lv, Di Liu, Xinmiao He, Wentao Wang, Yanzhong Feng, Md. Saiful Islam, Qiuju Wang, Wengui Chen, Ziguang Liu, Saihui Wu, Adam Abied

**Affiliations:** 1 Key Laboratory of Combining Farming and Animal Husbandry, Institute of Animal Husbandry, Heilongjiang Academy of Agricultural Sciences, Ministry of Agriculture and Rural Affairs, Harbin, P. R. China; 2 Wood Science Research Institute of Heilongjiang Academy of Forestry, Harbin, P. R. China; 3 Department of Animal Production & Management, Sher-e-Bangla Agricultural University, Sher-e-Bangla Nagar, Dhaka, Bangladesh; 4 Key laboratory of Heilongjiang Soil Environment and Plant Nutrient, Institute of Soil Fertilizer and Environment Resources, Heilongjiang Academy of Agricultural Sciences, Harbin, P. R. China; 5 Animal Science and Technology College, Northeast Agricultural University, Harbin, P. R. China; 6 Dry Land Research Center (DLRC) and Animal Production, Agricultural Research Corporation (ARC), Khartoum, Sudan; 7 Projects and Programs Secretary of the Sudan Youth Organization on Climate Change, Khartoum, Sudan; Government College University Faisalabad, Pakistan, PAKISTAN

## Abstract

The processes involved in soil domestication have altered the soil microbial ecology. We examined the question of whether animal manure application affects the soil microbial ecology of farmlands. The effects of global animal manure application on soil microorganisms were subjected to a meta-analysis based on randomized controlled treatments. A total of 2303 studies conducted in the last 30 years were incorporated into the analysis, and an additional 45 soil samples were collected and sequenced to obtain 16S rRNA and 18S rRNA data. The results revealed that manure application increased soil microbial biomass. Manure application alone increased bacterial diversity (M-Z: 7.546 and M-I: 8.68) and inhibited and reduced fungal diversity (M-Z: −1.15 and M-I: −1.03). Inorganic fertilizer replaced cattle and swine manure and provided nutrients to soil microorganisms. The soil samples of the experimental base were analyzed, and the relative abundances of bacteria and fungi were altered compared with no manure application. Manure increased bacterial diversity and reduced fungal diversity. *Mrakia frigida* and *Betaproteobacteriales*, which inhibit other microorganisms, increased significantly in the domesticated soil. Moreover, farm sewage treatments resulted in a bottleneck in the manure recovery rate that should be the focus of future research. Our results suggest that the potential risks of restructuring the microbial ecology of cultivated land must be considered.

## Introduction

Arable land is under unprecedented pressure due to the continual increases in global population and food demand [1–3]. Forests and grasslands have been reclaimed as cultivated land to obtain more grain. With the development of agricultural activities, soil has gradually become domesticated [4]. Soil and rhizosphere microbial communities can affect plant growth. Thus, in order to produce more food, the soil microbial ecology has been altered due to soil domestication [5, 6].

The soil microbial community is an important natural carbon sink and plays important roles in the carbon cycle and in greenhouse gas emissions (S1 Fig) [6, 7]. Soil microbial communities have the capacity to maintain themselves in terms of stability and self-healing abilities in case they are damaged or destroyed [8]. Manual application is a process of enrichment of N, P, K, and carbon. When applied microbial communities are combined with a natural soil microbial communities, an ecological balance will be reestablished through competition. Many studies have been conducted concerning the effects of animal manure application on soil microorganisms in farmlands [9, 10]. Manure has its own microbial community [11] and is a traditional fertilizer that has been used for thousands of years. In recent years, animal manure has no longer been the most commonly used type of fertilizer. In the carbon cycle, refractory veterinary drugs used to prevent and control animal diseases enter the soil, leading to soil pollution. Additives and heavy metals in animal feed also enter the soil and affect the normal growth and development of plants as well as and crop yields [12]. Under these conditions, manure use should be limited.

Soil microorganisms have been divided into five groups: soil bacteria, soil actinomycetes, soil fungi, soil algae, and soil protozoa [13]. Some studies suggest that manure application can improve soil microbial biomass carbon (MBC), while others suggest that it can reduce soil MBC [14, 15]. A recent meta-analysis found that the soil microbial biomass in China has changed due to manure application [16]. However, it is necessary to conduct a meta-analysis at a global scale to investigate the effects of animal manure application on soil microorganisms.

Meta-analysis is a useful technique for analyzing and summarizing results of studies focused on the same field [17, 18]. In this meta-analysis, two independent analyses based on random control treatments were used. Soil MBC can be used to measure soil microbial biomass, and Shannon’s diversity index (i.e., Shannon–Wiener or Shannon–Weaver) can be used to measure soil microbial diversity. A trial sequential analysis (TSA) was used to determine the reliability of our findings. To study the effect of fertilization on soil microorganisms, the changes in the abundance of different microorganisms in the microbial community were assessed. We collected soil samples from the demonstration base and performed a verification experiment. These results will serve as a reference concerning the effects of animal manure application on the community structure of soil microorganisms, and the findings will provide theoretical guidance for the sustainable development of agriculture and ecological protection at a global scale.

## Materials and methods

### Database search strategy and data extraction

Two independent meta-analyses systematically searched the following databases for studies published from January 01, 1990 to July 01, 2020: Ovid, Proquest, ScienceDirect, and Google Scholar. The following search terms were used in the MBC meta-analysis: (animal OR pig OR hog OR swine OR porcine OR cattle OR cow OR bovine OR poultry OR chicken OR sheep OR goat OR horse OR livestock) AND (manure OR compost OR mud OR sludge OR ooze OR effluent OR waste OR dung OR slurry) AND ((microbial biomass carbon) OR MBC). For the soil microbial diversity meta-analysis, the following search terms were used: ((Shannon index) OR (Shannon–Weaver index)) AND ((microbial community) OR (bacterial community) OR (fungal community)). The study inclusion and exclusion criteria are described in Table 1.

**Table 1:** Inclusion and exclusion criteria.

Inclusion	Exclusion
Randomized controlled trials (RCT)	Not RCT
English	Non-English
Control group included (M–Z or M–I)	No control group
For the microbial biomass carbon (MBC) meta-analysis: MBC data included	No MBC data
For the soil microbial diversity meta-analysis: Shannon data included	No Shannon data
Original research	Review

The included studies consisted of different comparisons with manure application; thus, the control groups were considered zero fertilization (Z) and/or inorganic fertilization (I). Other fertilization conditions were abbreviated as manure (M), manure plus inorganic fertilization (MI), and fallow (F). Seven dataset types were defined in this study: MBC response to manure application compared to an unfertilized control (M–Z) and six other groups: I–Z (inorganic fertilization application compared to an unfertilized control); MI–Z (manure plus inorganic fertilization compared to an unfertilized control); M–I (manure fertilization compared to an inorganic fertilization control); MI–I (manure plus inorganic fertilization compared to an inorganic fertilization control); MI–M (manure plus inorganic fertilization compared to a manure control), and F–Z (fallow compared to an unfertilized control) [19]. Each dataset group was extracted separately and analyzed as independent data. The Shannon index represents the diversity of microorganisms, and the Shannon index based on 16S or 18S sequencing represents the relative abundance of microorganisms. Therefore, the Shannon index based on 16S or 18S sequencing was not considered in our meta-analysis.

### Data analysis

There are many methods for MBC content measurement. The fumigation increment method and the fumigation extraction method are the most commonly used, but the results can be quite different. Thus, the present study used the MBC change rate (MBCR) to estimate the change in value [20]. The MBCR for each of the seven datasets was calculated as follows:

$$\bar{MBCR}=\sum_{i=1}^{n}\left(\frac{{MBC}_{M}-{MBC}_{C}}{{MBC}_{C}}\right)\times \frac{1}{n}\times 100\%,$$

where MBC_M_ is the MBC of manure application, and MBC_C_ is the control group. Standard errors (SE) were calculated as follows:

$$SE=\frac{\sqrt{\sum_{i=1}^{n}{\left[{\left(\frac{{MBC}_{M}-{MBC}_{C}}{{MBC}_{C}}\right)}_{i}-\bar{MBCR}\right]}^{2}}}{n}.$$

Review Manager v5.3 (Nordic Cochrane Centre, Copenhagen, Denmark) and R v3.2.2 software (R Development Core Team, Auckland, New Zealand) were used for the meta-analyses. A TSA was used to evaluate the reliability of the data and was conducted using Trial Sequential Analysis Viewer (Copenhagen Trial Unit, Copenhagen, Denmark).

Shannon index changes were calculated using the same method as that used for MBCR. When the bacterial data and fungal Shannon index changes were present in the same literature and when the change value did not conform to a normal distribution, a Mann–Whitney *U* test was used to detect differences between groups using SPSS software (SPSS Inc., Chicago, IL, USA). The effects of manure on yield and MBCR were calculated by regression analysis. Since the application amount of manure in our study did not exceed 5 T/ha, a maximum application amount of 30 T/ha was applied in the calculations.

We analyzed the effects of manure on MBCR according to the type of manure. This study also attempted to determine the relationship between each factor and MBCR increase through a network meta-analysis or regression analysis, but it became obvious that the application rate was the most important factor; therefore, an analysis of the application rate along with soil conditions such as pH and depth was unnecessary.

### Soil sample collection and analysis

To study the effect of fertilization on soil microorganisms, the change in abundance of different microorganisms in the microbial community was assessed. Forty-five soil samples were collected from Qingfeng village, Tangyuan county, Jiamusi city, Heilongjiang Province, and from the combined farming and animal husbandry demonstration base of the Animal Husbandry Research Institute, Heilongjiang Academy of Agricultural Sciences (longitude 129.68, latitude 46.67). The site is situated at an altitude of 79 m, with an annual precipitation of 520 mm, an average annual temperature of 3°C, and black soil. Sampling commenced on 21 November 2020. The cultivation conditions included rotary tillage to 20 cm with a mixture of pig manure and chicken manure. Five types of non-rhizosphere soil, uncultivated U, Z, I, M, and MI, were collected and applied for three to six years. The sampling depths were 0–10 cm, 10–20 cm, and 20–30 cm, and each sampling plot included three different locations. The grouping method was as follows: U01 represents U0–10 cm, U02 represents U10–20 cm, U03 represents U20–30 cm, and the other groups followed the same pattern.

The soil samples were frozen in liquid nitrogen in a centrifuge tube. Soil DNA was extracted using a Soil DNA Extraction Kit (Omega, Doraville, GA, USA), and the quality of the extracted DNA was assessed for the construction of 16S and 18S rDNA libraries. The PCR products were purified by Agencourt AMPure XP beads (Beckman Coulter, Indianapolis, IN, USA) and quantified by a PicoGreen double-stranded DNA quantitative detection kit (Invitrogen, Carlsbad, CA, USA). The V3-V4 hypervariable region of the 16S rDNA and V4 region of 18S rDNA were detected by an Illumina NovaSeq6000 sequencing platform to analyze the community structure of the soil microorganisms.

After the original sequencing data were obtained, clean data were obtained by sequence filtering and splicing, and operational taxonomic unit (OTU) clustering was conducted using USEARCH software (v9.2.64). The sequence with the highest abundance was selected from the same OTU as the representative sequence of the OTU. For bacteria and fungi, QIIME (v1.9.1) software was used to compare the representative sequences with the Silva 132 and PR2 (v4.12.0) databases to obtain species annotation information. Welch’s *t*-test was used to compare the species differences between groups. R software (v3.6.0) was used for correlation analysis of species abundance, and Cytoscape software (v3.6.0) was used for mapping.

## Results

**Fig 1:**
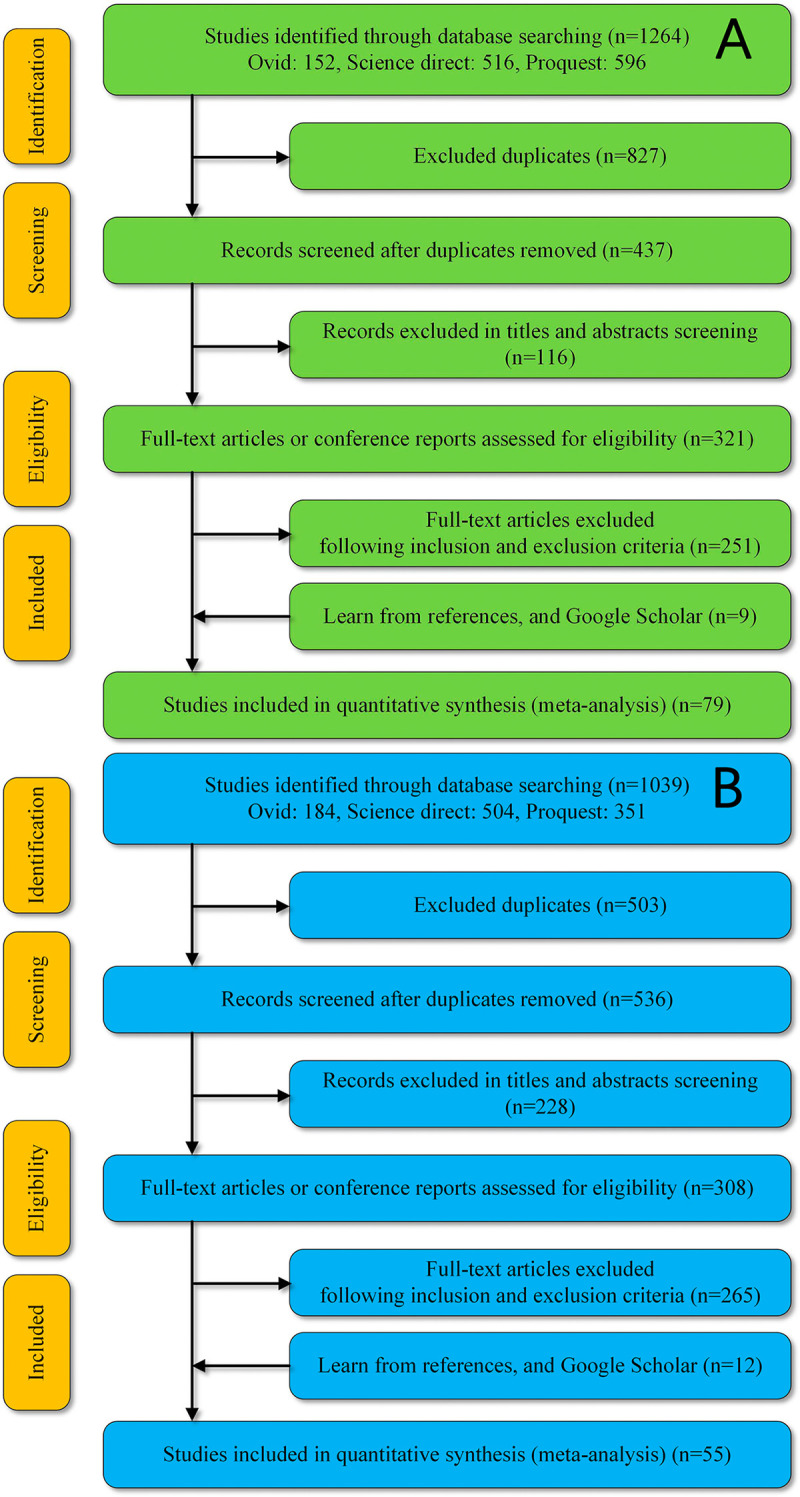
Summary of the study selection processes for MBC (A) and Shannon index (B) meta-analyses.

**Fig 2:**
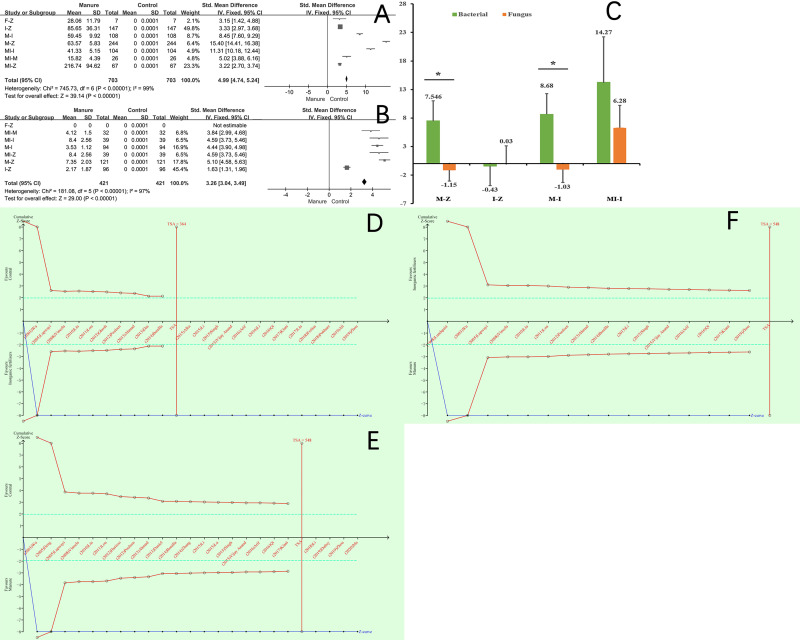
Forest plot of the effects of manure on microbial biomass carbon (MBC) (A) and the Shannon index (B). Confidence interval (CI) = 95%. Mann–Whitney *U* test of the Shannon index (C). * indicates a significant difference (*p* < 0.05). Trial sequential analysis (TSA) of the effects of manure on I-Z (D), M-Z (E), and M-I (F).

Initially, a total of 2303 studies (MBC, 1264; soil microbial diversity, 1039) were found from the literature search. The study selection process is presented in Fig 1A and 1B. Ultimately, 134 reports (MBC, 79; soil microbial diversity, 55) and 871 datasets (MBC, 703; soil microbial diversity, 168) were included in the meta-analysis. The characteristics of all of the included studies are provided in S1 and S2 Tables. Our results found that seven datasets showed improved MBCR (Fig 2A).

A total of six merged datasets showed improved microbial diversity of the soil (Fig 2B). Interestingly, the application of manure alone increased bacterial diversity (M-Z: 7.546 and M-I: 8.68) as well as inhibited and reduced fungal diversity (M-Z: −1.15 and M-I: −1.03) (Fig 2C). The TSA indicated that the analysis results were reliable (Fig 2D–2F). The TSA also showed that the results of the manual effect MBC test exceeded the threshold value, and there was no need to conduct further manual effect MBC tests in the future. Thus, it was determined that the soil microbial ecology was altered during the soil domestication process.

**Fig 3:**
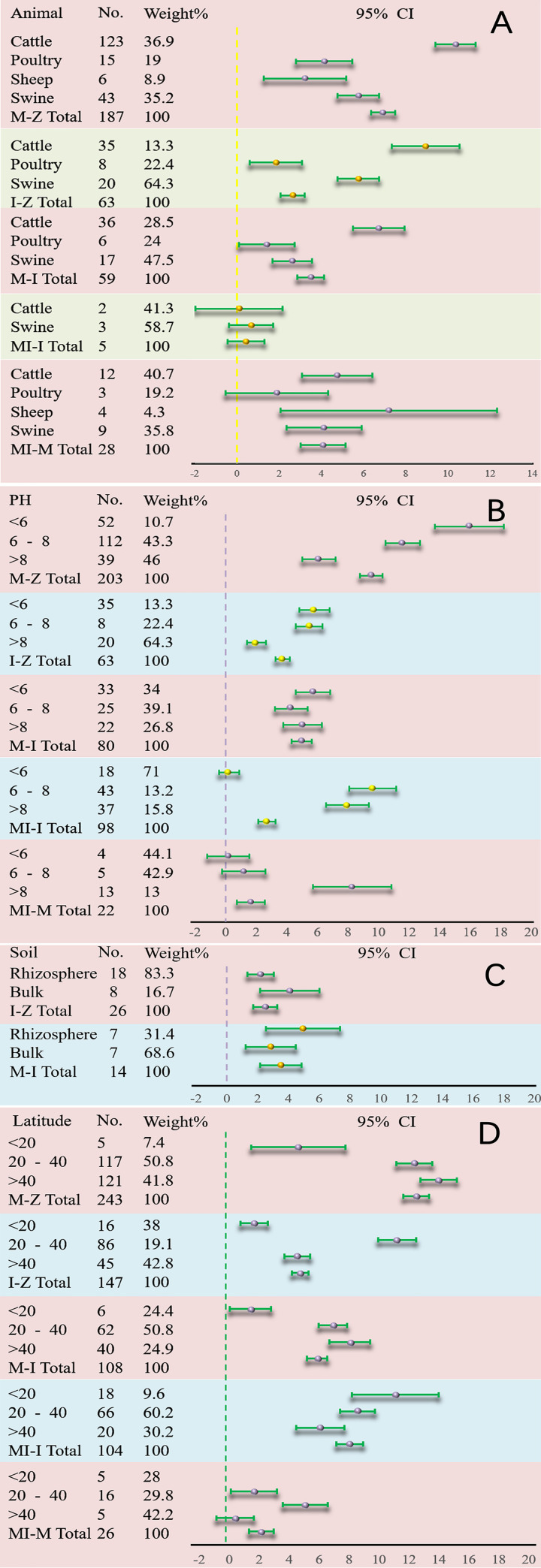
Forest plot based on effects of different factors on MBC: type of manure (A), pH (B), rhizosphere soil (C), and latitude (D). CI = 95%.

The effects of different factors on MBCR were also investigated. The examined factors were: the type of manure, pH, rhizosphere and bulk soils, latitude, texture, rotation, tillage, depth, duration, and application rate. The majority of the four dataset results indicated that the test group increased MBCR compared to the control group (Fig 3A–3D). All of the results mentioned above were possibly affected through publication bias; therefore, all of the results should be treated with caution.

**Fig 4:**
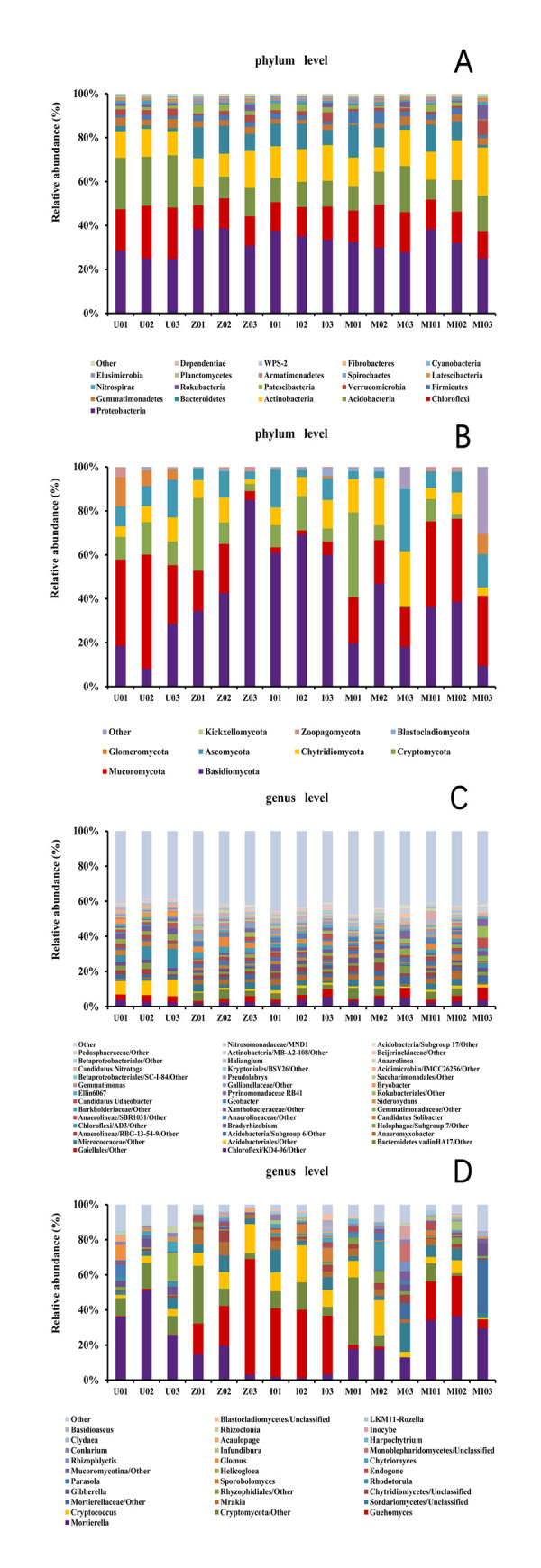
Microorganism composition of the five soil types. Bacterial phylum level (A), fungal phylum level (B), bacterial genus level (C), and fungal genus level (D).

At the phylum level, the top five bacterial phyla exhibited little change among the experimental groups, while the fungi had relatively significant changes. The top five bacterial and fungal taxa at the genus level differed significantly among the experimental groups (Fig 4A–4D).

**Fig 5:**
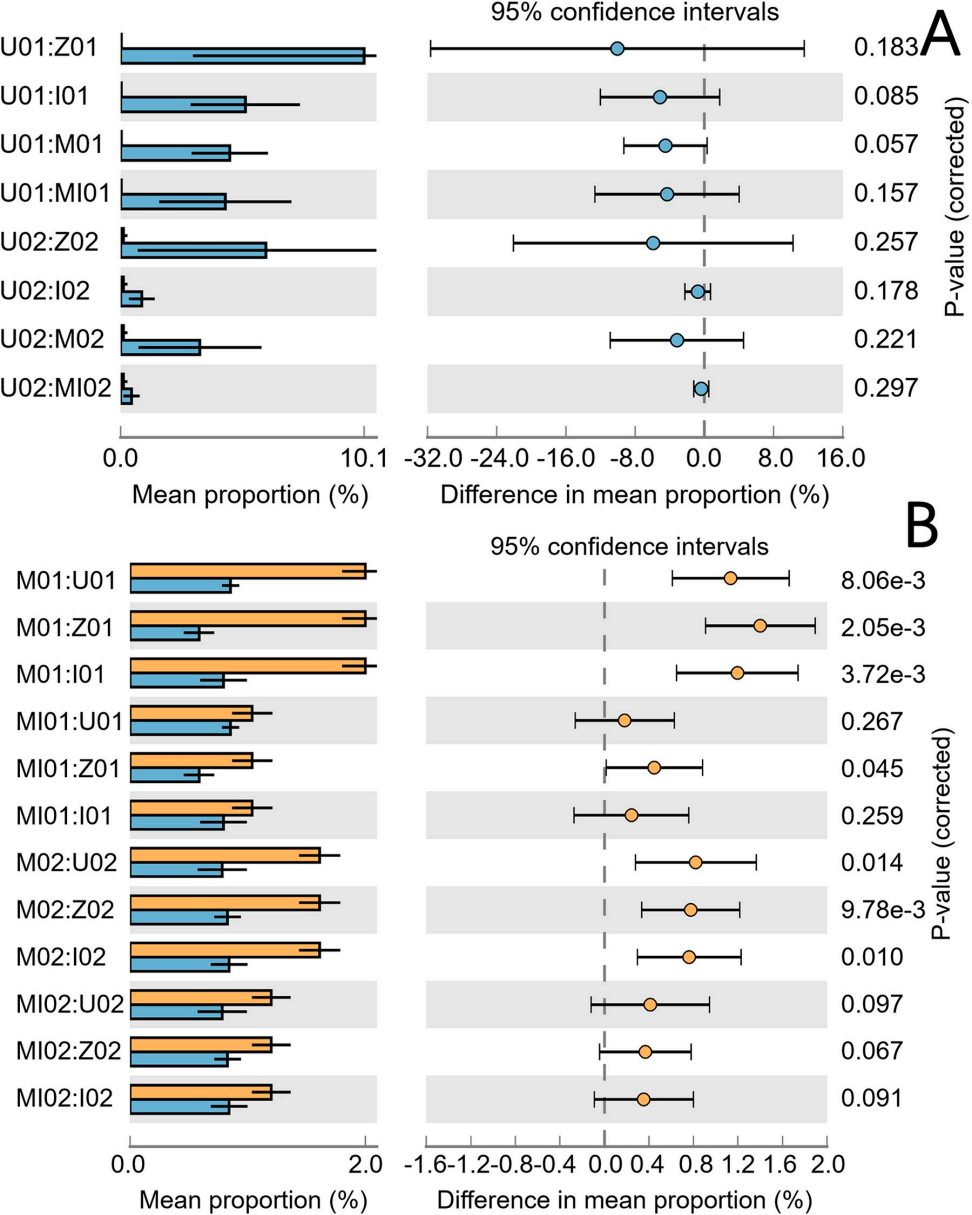
Inter-group difference test of microorganism relative abundance. *Mrakia frigida* (A), *Betaproteobacteriales* (B).

In particular, the abundances of *Anaeromyxobacter*, *Anaerolineaceae*, *vadinHA17* (S2A–S2C Fig) and *Mrakia frigida* (Fig 5A) were lower in U01 and U02 than in the other groups. In addition, the abundance of *Endogenone* in U02 was lower than that in the other groups (S2D Fig). The abundances of *Xanthobacteraceae* and *Bryobacter* in U01 and U02 were higher than in the other groups (S2E and S2F Fig). Interestingly, *Burkholderiaceae*, which causes disease in gramineous plants, also increased in the soil under land reclamation (S2G Fig). In addition, *Betaproteobacteriales* increased significantly in the soil supplied with manure (Fig 5B).

## Discussion

Land management is one of the biggest factors contributing to soil production and climate change. In addition, one of the core components of sustainable agricultural development is the carbon cycle [20]. In this cycle, animal manure applied to farmland is the main source of crop nutrients [21].

Swine manure is rich in proteins and fats and easy to decompose. It contains several humic substances with high water content [22, 23]. The nutrient content of cattle manure is low; it decomposes slowly and has poor air permeability and so it is not used as poultry manure [22, 23]. Sheep (goat) manure has good texture and contains more organic compounds than other animal manure. It is a neutral fertilizer suitable for sandy soil and clay [24]. Poultry manure is rich in nitrogen (N) but contains parasite oocytes and heavy metals [22, 23].

All fertilizers can be used in fermentation treatments. In this study, two independent analyses based on randomized controlled treatments were used to measure soil microbial biomass and Shannon diversity index. Our results revealed that there were no differences in the sources of cattle manure and swine manure in the MI–I group, indicating that inorganic fertilizer can completely replace cattle and swine manures and provide nutrients to soil microorganisms. There was no difference in poultry manure in the MI–M group, indicating that poultry manure had a unique promoting effect on soil microorganisms.

### Factors affecting manure efficiency

In terms of soil depth, the content of soil total nitrogen and organic carbon increased after the application of organic fertilizer [25]. Based on our field experience, the average application rate of manure is 1–3 T/ha. The density is estimated to be 1, which is equivalent to an increase of 0.2 mm in the soil layer. This is much faster than the natural formation rate of soil. Of course, the annual production of grain and fertilizer and output of cultivated land are also included in the calculations of land carrying capacity. It is generally believed that there are fewer microorganisms in the plow pan than in the plow layer. Our results revealed that the MBCR increased at all depths. Under the same volume, different soil textures have different surface areas, so the attached bacteria are completely different, resulting in different MBC contents [14]. Compared with inorganic fertilizer, manure increases water-stable aggregates [25]. The application rate is the key factor affecting MBC; different application rates resulted in different MBC values.

Plant roots can be used as a channel for material exchange [26, 27]. Rhizosphere and bulk soils contain different amounts of MBC. Fertilization can improve the decomposition rate of roots [28]. There is a mutualistic relationship between soil microorganisms and plants [29]. Rhizosphere microbial ecology can affect the secretion of plant root metabolites [30], and plants can also affect rhizosphere microbial activities [5]. Rice (*Oryza sativa* L.) planting can change the soil microbial ecology due to the secretions and metabolism of rice plants [4]. Specific metabolites produced by *Arabidopsis thaliana* can selectively change the microbial community in rhizosphere soil to meet the needs of plants [31].

In different latitudes, the effects of temperature are different, and the activities of microorganisms are also different [32]. Tillage farming is a traditional agricultural method, and currently, there have been many studies on no-tillage management [33]. In the process of tillage, the soil is disturbed and the porosity changes accordingly. Although no-tillage soil fertilizer is placed below the seeds, the porosity of no-tillage soil is poor, resulting in the reduction of fertilizer nutrient diffusion.

Crop rotation is a common farming management method. Our farm rotates soybeans (*Glycine max* L.) once every few years, as soybeans can improve soil fertility. A previous study showed that the continuous monoculture of rice would affect soil microorganisms [4]. MBC is also different due to different crops [14]. Groundnuts have rhizobia, and recent studies have found that, similar to legumes, native *Z*. *mexicana* varieties can fix nitrogen without fertilization [34].

We found that the abundance of *Xanthobacteraceae* in uncultivated land was higher than in the other groups. *Xanthobacteraceae* tends to utilize natural nitrogen sources [35]. After domestication, the proportion of bacteria in this family decreased with the increase of fertilization. When we collected samples from paddy fields, the abundances of three anaerobic bacteria, *Anaeromyxobacter*, *Anaerolineaceae*, and *vadinHA17*, were higher than in the uncultivated land. Due to rice cultivation, *Burkholderiacea*e was also more abundant in the uncultivated land [36].

Our previous studies have shown that feed can change the intestinal flora of animals [37]. Another study showed that the dominant intestinal microflora could be detected in manure [11]. Without fermentation treatment, manure microorganisms enter the biosphere and may cause animal diseases. In the process of manure fermentation, temperatures can reach 70°C so as to reconstruct the microbial community, and this effect is closely related to pH [38]. Manure maintains soil pH, while inorganic fertilizer can lead to soil acidification [25].

Soil microbial metabolism not only affects the material circulation and ecological balance but also promotes soil fertility and nutrient transformation in plants [7, 28]. The changes in soil microbial metabolism are influenced by carbon fixation [39], nutrient acquisition [40], decomposition, soil enzyme activity [41], soil formation [42], and carbon and nitrogen cycling [39, 43].

### The importance of soil microorganisms to agriculture

Microorganisms play an important role in improving soil fertility, crop characteristics, and grain yield as well as controlling diseases and pests. *Piriformospora indica* protects the growth of tomato under salt stress [44]. The combination of *Pseudomonas* and *Bacillus* improves the health of plants [45]. Beneficial soil microorganisms improve soil health, promote plant growth, and finally promote the sustainable development of agriculture [8]. Researchers have recognized the need for beneficial microorganisms [46]. Improving soil beneficial microorganisms is one of the approaches for soil remediation and improvement. Compared with manure, this will reduce the damage to the soil microbial community. Our results showed that the addition of manure improved soil microbial diversity and increased microbial biomass; however, highly active soil microorganisms in soil carbon sinks will be reduced [47].

Applying fertilizers (M–Z and M–I) alone increases bacterial diversity, and inhibits and reduces fungal diversity. Compared with animals and plants, soil microorganisms are more likely to evolve with environmental changes. Interestingly, soil microorganisms have evolved their own competitive characteristics. Due to the limitation of soil migration, it is difficult for microorganisms to disperse far away. *Streptomyces* colonies that release a unique odor attract springtails that can transport the microorganisms to a distant location [48]. Interestingly, our results showed that the relative abundance of *Mrakia frigida* is low in uncultivated land. *Mrakia frigida* can inhibit the growth of other fungi [49]. *Betaproteobacteriales* in soil increased significantly and inhibited the growth of other microorganisms [50].

### Relationship between soil microbial ecology and human ecology

Soil microbial ecology is closely related to the global climate [6, 51]. Climate warming will lead to a series of problems, and greenhouse gases are the leading causative factor [52]. Several studies have indicated that the application of inorganic fertilizers contributes to greenhouse gas emissions. Mining and the transporting inorganic fertilizers can also lead to carbon emissions. The use of inorganic fertilizer may lead to increased soil acidification and phosphorus (P) uptake [53], thereby increasing greenhouse gas emissions [47].

However, our study found that this may not be the case. Application of inorganic fertilizer and manure increased MBC, but inorganic fertilizer did not contain a carbon source. Most of the increased MBC is absorbed from the gas and soil as a carbon source. Hyperactive soil microorganisms decrease terrestrial carbon sinks [47]; therefore, it seems that increasing the MBC of agricultural activities will reduce carbon sinks. Other studies have shown that both organic and inorganic fertilizers should be properly evaluated; otherwise the carbon sinks of soil carbon pools will be incorrectly evaluated [28]. Fresh manure cannot be directly applied to farmland. Many greenhouse gases will be produced in the process of manure fermentation, resulting in energy waste. Therefore, the effect of greenhouse gas emission may not significantly differ from that of inorganic fertilizer.

### Farm manure treatments

Animal husbandry produces about 4.6 billion tons of fertilizer every year. However, due to the high labor cost of collecting, transporting, and transferring manure to farmland and the lack of appropriate storage and treatment facilities, only a small portion of manure fertilizer is applied to farmland [16, 54]. The TSA verified the reliability of our results and showed that previous reports on fertilizer increasing soil microorganisms are valid. Future studies should focus on how to reduce costs and improve the use of manures. For example, some countries have enacted laws requiring the use of anti-seepage septic tanks for manure application. This study also suggested that the field manure application should be enforced through legislation, although this will undoubtedly increase the cost of livestock products, and consumers will eventually bear the burden of this cost. First, it should be ensured that grain production meets demand. Second, environmental protection and sustainable development should be considered. The large-scale development of agriculture is the trend of future development. Cultural enterprises can carry out manure fermentation treatments. However, some farms in some areas do not have the proper fermentation conditions and depend on assimilation capacity of land, while farmland accumulation depends on natural fermentation.

We do not encourage the use of inorganic fertilizer, but advocate the use of fertilizers. This study advocates combining inorganic fertilizer and organic fertilizer based on soil and environmental factors. Therefore, it is recommended to make rational use of biogas [54], incineration, and power generation [55]. Biogas and circular agriculture can be carried out in temperate regions. However, in cold regions, biogas production is difficult, especially in winter. Therefore, more research on manure incineration and power generation should be carried out in cold regions. In tropical areas, the removal of heavy metals in septic tanks requires the use of a chelating agent or electrode. The contents of N and P in manure are high, so it is difficult to meet crop demands by applying fertilizer alone. Inorganic fertilizer is applied under the seed, while fertilizer is applied by spreading. With the large-scale agricultural mechanization, fertilizer application has gradually lost its competitiveness [56].

Every crop has its own form of nutrient guidance. The application of inorganic fertilizer can be completed during sowing, and the effect is rapid [16]. At least two manual steps should be added for fertilizer application, but these will eventually increase costs. Environmental protection and sustainable development are the starting points of many studies [20], but the economy is the key factor in market dynamics. Farmers believe that the application of inorganic fertilizer will produce higher yields than that of organic fertilizer [16]. In addition, the cost of fertilizer is high. Only when farmers are less busy in winter can they get manure from animal factories and send them to farmland. Therefore, inorganic fertilizer can be applied in spring season.

### Bottlenecks and other issues for future investigations

The recovery rate of fertilizer is not high, and many studies have focused on its application [38, 42]. A large amount of wastewater is produced in farm cleaning and culturing [57, 58]. At present, there is no reasonable treatment method (manure recovery), and the known methods include spraying foliar fertilizer or recycling green algae [59]. However, the cost of these treatments is high, thus limiting the efficiency of recovery. China has introduced policies for restricting pig breeding in warm areas in the south and moving pig farms northward. An important reason for this is that aquaculture sewage pollutes drinking water sources [60]. Efforts have also been made to reduce the widespread use of inorganic fertilizer in cold regions in the north. These two goals have been gradually achieved. The cold winter in the north does not affect manure fermentation. As can be seen from the world map, 46% of the reports in this meta-analysis are from China and 18% are from India, indicating that developing countries are more likely to face environmental problems in agricultural development [56]. Therefore, these issues should be considered and investigated in future studies.

## Conclusions

The use of manure in farmland increases bacterial diversity, reduces fungal diversity, and destroys the ecological balance. Of course, this change is much smaller than the use of probiotics. Although soil domestication will lead to the destruction and reconstruction of the soil microbial community, the affected soil microorganisms will increase grain production to meet human food needs. Although these results improve our well-being, our results suggest that the potential risks of rebuilding the microbial ecology of cultivated land must be considered.

## Supporting information

S1 Checklist (DOCX)

S1 Fig Effects of manure on the soil microbial ecology and graphical abstract. The planting industry provides feed for animals, and animal manure is applied to farmland. In such a cycle, we obtain grain and meat products. Veterinary drug, feed additives, pesticide, inorganic fertilizers, and plant hormones are used in this cycle. This will affect soil microbial ecology. Manure when applied to farmland increased bacterial diversity as well as reduced fungal diversity. The use of manure destroyed the ecological balance in farmland.
(TIF)

S2 Fig Inter-group difference test of microorganism relative abundance. Anaeromyxobacter (A), Anaerolineaceae (B), VadinHA17 (C), Endogone (D), Xanthobacteraceae (E), Bryobacter (F), Burkholderiaceae (G).
(TIF)

S1 Table Characteristics of the MBC studies included in the meta-analysis. (DOCX)

S2 Table Characteristics of the Shannon index studies included in the meta-analysis. (DOCX)
