# Supplementary figures and images for: A global meta-analysis of animal manure application and soil microbial ecology based on random control treatments
Source: PLoS One. 2022 Jan 21;17(1):e0262139. doi: 10.1371/journal.pone.0262139 (PMC8782357; doi:10.1371/journal.pone.0262139)

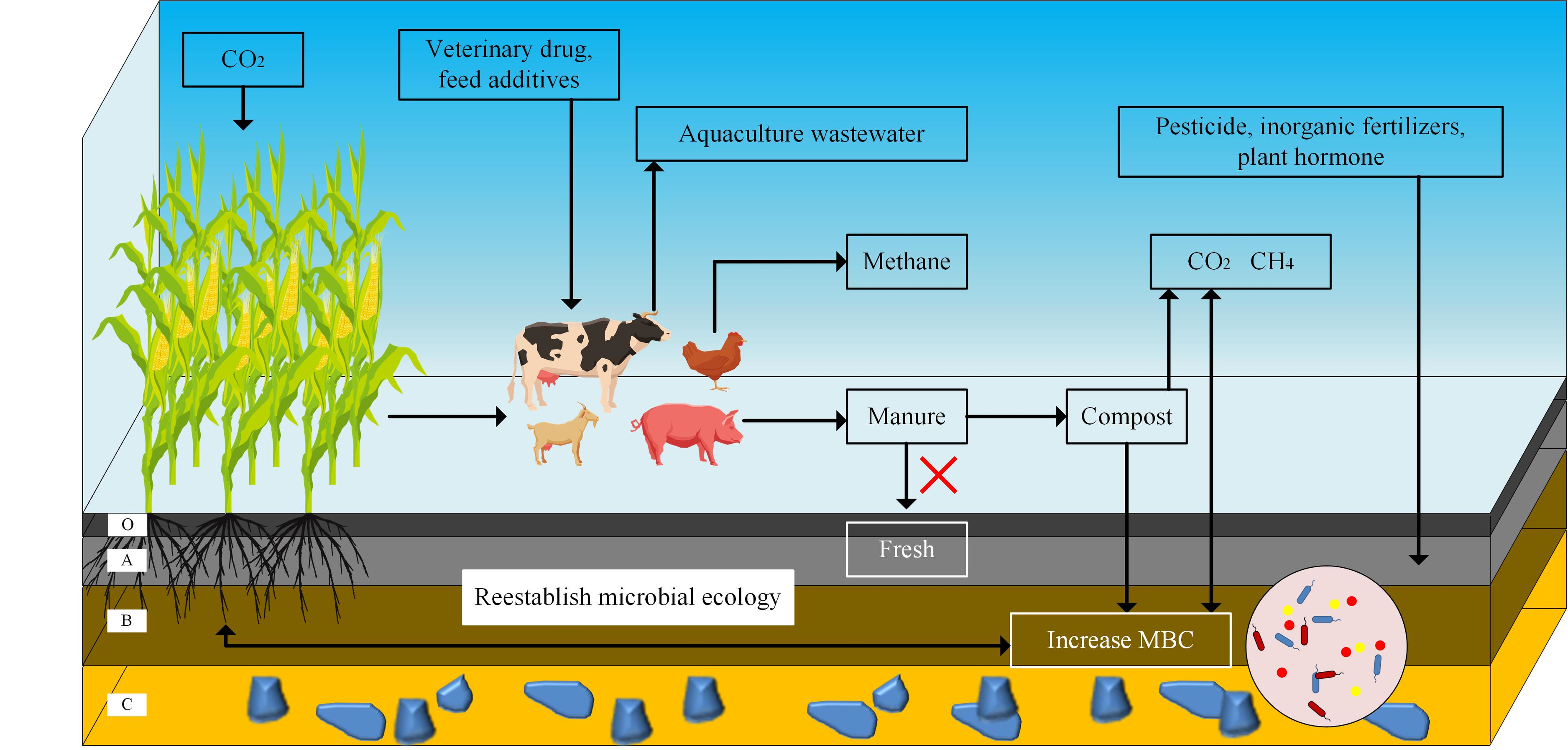

Supplement: S1 Fig — The planting industry provides feed for animals, and animal manure is applied to farmland. In such a cycle, we obtain grain and meat products. Veterinary drug, feed additives, pesticide, inorganic fertilizers, and plant hormones are used in this cycle. This will affect soil microbial ecology. Manure when applied to farmland increased bacterial diversity as well as reduced fungal diversity. The use of manure destroyed the ecological balance in farmland. (TIF) [file pone.0262139.s002.tif]

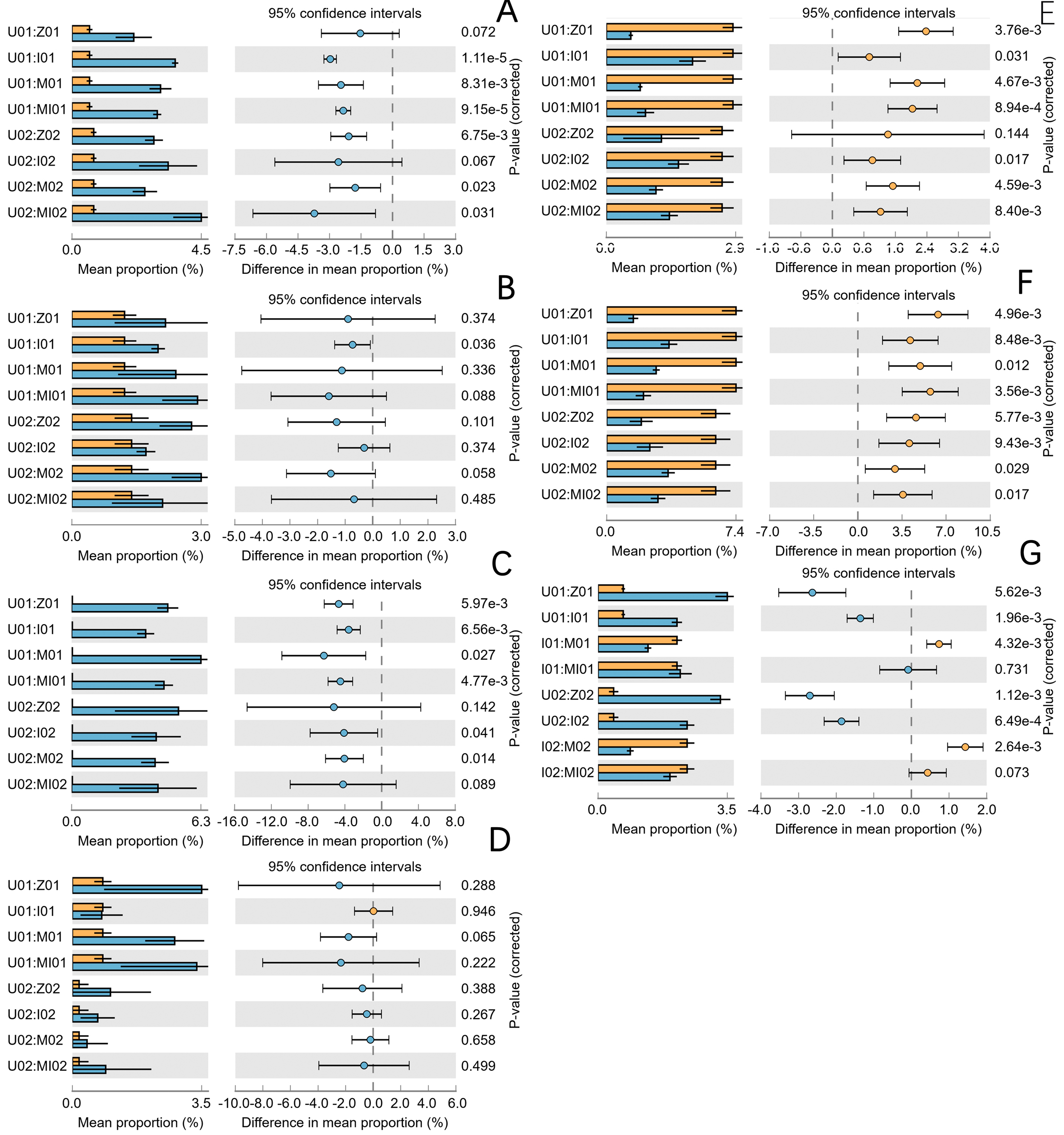

Supplement: S2 Fig — Anaeromyxobacter (A), Anaerolineaceae (B), VadinHA17 (C), Endogone (D), Xanthobacteraceae (E), Bryobacter (F), Burkholderiaceae (G). (TIF) [file pone.0262139.s003.tif]
