# Supplementary material for: A global meta-analysis of animal manure application and soil microbial ecology based on random control treatments
Source: PLoS One. 2022 Jan 21;17(1):e0262139. doi: 10.1371/journal.pone.0262139 (PMC8782357; doi:10.1371/journal.pone.0262139)
Supplement: S1 Table — (DOCX) [file pone.0262139.s004.docx]

Table S1. Characteristics of the MBC studies included in the meta-analysis.

| No. | Study ID | Data set  No. | Dataset group | Location | Latitude and longitude | Annual  precipitation  (mm) | Annual average  temperature (℃) | Depth (cm) | Duration |
| --- | --- | --- | --- | --- | --- | --- | --- | --- | --- |
| 1 | Lundquist, 1998[[1](#_ENREF_1)] | 4 | M-I | California USA | 32.16°N, 110.85°W | NM | NM | 0-2  2-15 | 3 years |
| 2 | Kanchikerimath, 2000[[2](#_ENREF_2)] | 1 | I-Z, MI-I | New Delhi, India | 28.40°N, 77.10°E | 670 | 24 | 0-15 | 30 years |
| 3 | Wu, 2003[[3](#_ENREF_3)] | 1 | M-Z, M-I, I-Z, MI-M, MI-I | Gansu, China | 36.03°N, 103.40°E | 560 | 10.2 | 0-20 | 20 years |
| 4 | Manna, 2004[[4](#_ENREF_4)] | 10 | M-Z, M-I, I-Z, MI-M, MI-I | Barrackpore, India  Ranchi, India  Akola, India | 22.75°N, 88.43°E  22.5°N, 85.25°E  20.70°N, 77.03°E | 1698  1450  824.7 | NM | 30 | 29 years  30 years  14 years |
| 5 | Bhattacharyya,.2005[[5](#_ENREF_5)] | 20 | M-Z, M-I, I-Z | Baruipur, India | 22.33°N, 88.43°E | NM | 23.5 | 0-30 | 1-3 years |
| 6 | Deng, 2005[[6](#_ENREF_6)] | 6 | M-Z | Oklahoma, USA | 40.32°N, 81.3°W | 447 | 13.8 | 0-10 | 10 years |
| 7 | Lupwayi, 2005[[7](#_ENREF_7)] | 14 | M-Z, M-I, I-Z | Falher Alberta, Canada  Fairview Alberta, Canada | 55.72°N, 117.2°W  56.06°N, 118.38°W | 367  223 | NM | 0-15 | 1-3 years |
| 8 | Masto, 2005[[8](#_ENREF_8)] | 1 | I-I | New Delhi, India | 28.63°N, 77.17°E | 670 | 23.5 | 0-15 | 31 year |
| 9 | Pal, 2005[[9](#_ENREF_9)] | 14 | M-Z | Mohanpur, India  West Bengal, India | 22.87°N, 88.50°E  22.25°N, 88.67°E | NM | NM | 0-15 | 6 months |
| 10 | Purakayastha, 2005[[10](#_ENREF_10)] | 3 | I-Z, MI-I | New Delhi, India | 28.4°N, 77.1°E | 821 | 24 | 0-15  15-30  30-45 | 33 years |
| 11 | Manna, 2006 [[11](#_ENREF_11)] | 3 | I-Z, MI-I | Ranchi, India. | 23.50°N, 85.02°E | 1450 | 23.1 | 0-15  15-30  30-45 | 4 years |
| 12 | Diana, 2007[[12](#_ENREF_12)] | 3 | M-I | Toledo, Spain | 40.24°N, 3.41°W | 487 | 14 | 0-20 | 5 years |
| 13 | Pal, 2007[[13](#_ENREF_13)] | 1 | M-Z | West Bengal, India | 22.87°N, 88.50°E | 1300 | 23 | 0-15 | 2 years |
| 14 | Vineela, 2008[[14](#_ENREF_14)] | 5 | M-Z, M-I, I-Z, MI-M, MI-I | Andhra Pradesh, India  Karnataka, India  Karnataka, India  Tamil Nadu, India  Maharashtra, India | 14.68°N, 77.62°E  12.97°N, 77.58°E  16.5°N, 76.85°E  11.0°N, 77.0°E  17.68°N, 75.93°E | 643  924  632  612  742 | NM | 0-15 | 23 years  9 years  30 years  36 years  2 years |
| 15 | Gu, 2009[[15](#_ENREF_15)] | 2 | M-Z, M-I, I-Z, MI-M, MI-I | Sichuan china | 30.17°N, 105.05°E | 1014 | 17.5 | 0-20 | 1 year |
| 16 | Liu, 2010[[16](#_ENREF_16)] | 1 | M-Z, M-I, I-Z, MI-M, MI-I | Gansu, China | 35.27°N, 107.50°E, | 540 | 9.8 | 0-15 | 31 year |
| 17 | Abbasi, 2011[[17](#_ENREF_17)] | 1 | M-Z | Kashmir, Pakistan | 32.73°N, 74.87°E | NM | NM | 0-15 | NM |
| 18 | Li, 2011[[18](#_ENREF_18)] | 1 | M-Z, M-I, I-Z, MI-M, MI-I | Heilongjiang, China | 47.26°N, 126.38°E | 550 | 1.68 | 0-20 | 26 years |
| 19 | Lou 2011[[19](#_ENREF_19)] | 1 | M-Z, M-I, I-Z, MI-M, MI-I | Shenyang，China | 41.82°N, 123.57°E | 705 | 7.9 | 0-20 | 22 years |
| 20 | Nayak, 2011[[20](#_ENREF_20)] | 16 | MI-I | Ludhiana, India  Kanpur, India  Sabour, India  Kalyani, India | 30.56°N, 75.52°E  26.58°N, 80.34°E  25.14°N, 87.04°E  23.0°N, 89.0°E | 500  818  500  500 | 23 | 0-15  15-30  30-45  45-60 | 29 years  28 years  26 years |
| 21 | Yu, 2011[[21](#_ENREF_21)] | 5 | I-Z, MI-I | Hailun, China | 47.43°N, 126.63°E | 500-600 | 1.5 | 0-20 | 2 years |
| 22 | Biau, 2012[[22](#_ENREF_22)] | 2 | M-Z, M-I, I-Z | Gimenells, Spain | 41.65°N, 0.39°E | 192 | 19.1 | 0-30 | 8 years  9 years |
| 23 | Biswas, 2012 [[23](#_ENREF_23)] | 4 | M-Z, M-I, I-Z, MI-M, MI-I | New Delhi, India | 28.4°N, 77.1°E | 821 | 24 | 0-15 | 6 years |
| 24 | Ghosh, 2012[[24](#_ENREF_24)] | 1 | I-Z, MI-I | West Bengal, India | 23.0°N, 89.0°E | 1500 | 23.5 | 0-15 | 25 years |
| 25 | Hurisso, 2012[[25](#_ENREF_25)] | 6 | M-Z | Colorado, USA | 40.65°N, 04.98°W; | 330 | 11 | 0-5  5-15 | 3 years |
| 26 | Poulsen, 2012[[26](#_ENREF_26)] | 8 | M-I, M-Z, I-Z | Copenhagen, Denmark | 55.67°N, 12.30°E | NM | NM | 0-20 | 1 year |
| 27 | Ahmad, 2013[[27](#_ENREF_27)] | 6 | M-I, M-Z, I-Z | District Swabi, Pakistan | 34.15°N, 73.21°E | NM | NM | 0-20  20-40 | 3 days  6 days  10 days |
| 28 | Busaidi, 2013[[28](#_ENREF_28)] | 2 | M-Z | Rumais, Oman | 23.68°N, 57.98°E, | 109 | 27 | 0-20 | 2 years |
| 29 | Daniel, 2013[[29](#_ENREF_29)] | 9 | M-Z | Conill, Spain | 41.72°N, 1.45°E | 500 | NM | 0-5 | 3 years |
| 30 | Das, 2013[[30](#_ENREF_30)] | 8 | MI-I, I-Z  MI-M | Cuttack, India | 20.42°N, 85.92°E | NM | 27.3 | 0-15 | 1 year |
| 31 | Mandal, 2013[[31](#_ENREF_31)] | 4 | M-I, MI-I, M-Z | New Delhi, India | 28.4°N, 77.1°E | 651 | NM | 0-15  15-30 | 5 years |
| 32 | Manna, 2013[[32](#_ENREF_32)] | 2 | I-Z, MI-I | Madhya Pradesh, India | 23.12N, 79.57E | 1350 | NM | 0-30 | 40 years |
| 33 | Xu, 2013[[33](#_ENREF_33)] | 4 | MI-I | Xinjiang, China | 43.95°N, 87.46°E | 310 | 7.7 | 0-20 | 21 year |
| 34 | Bonilla, 2014[[34](#_ENREF_34)] | 2 | M-Z, M-I, I-Z | Senes de Alcubierre, Spain | 41.90°N, 0.5°W | 327 | 13.4 | 0-20 | 3 years |
| 35 | Elciol, 2014[[35](#_ENREF_35)] | 8 | M-Z | Parana, Brazil | 24.28°S, 53.83°W | NM | NM | 0-20 | 15 years |
| 36 | Luo, 2014[[36](#_ENREF_36)] | 1 | M-Z, M-I, I-Z, MI-M, MI-I | Liaoning Province, China | 40.80°N, 123.55°E | 629 | 7.5 | 0-20 | 32 years |
| 37 | Zhang, 2014[[37](#_ENREF_37)] | 2 | M-Z | Liaoning, Canada | 42.45°N, 122.47°E | 510 | 7.1 | 0-20  20-40 | 4 years |
| 38 | Alka, 2015[[38](#_ENREF_38)] | 2 | I-Z, MI-I | Varanasi India | 25.30°N, 83.02°E | NM | NM | 0-10 | 1 year |
| 39 | He, 2015[[39](#_ENREF_39)] | 3 | MI-I | Gongzhuling, China  Zhengzhou, China  Qiyang, China | 43.5°N, 124.8°E  26.75°N, 111.87°E  26.75°N, 111.87°E | 525  632  1250 | 4.5  14.3  18 | 0-20 | 17 years |
| 40 | Li, 2015[[40](#_ENREF_40)] | 1 | M-I, M-Z, I-Z | 德州 china | 36.83°N, 116.57°E | 569 | 13.4 | 0-20 | 25 years |
| 41 | Lu, 2015[[41](#_ENREF_41)] | 4 | M-Z | Henan, China | 34.53°N, 115.50°E | 777 | 13.9 | 0-10 | 2 years |
| 42 | Singh, 2015[[42](#_ENREF_42)] | 1 | M-I, M-Z, I-Z | West Bengal, India | 22.75°N, 88.43°E | 1550 | 26.1 | 0-15 | 2 years |
| 43 | Vijay Anand, 2015[[43](#_ENREF_43)] | 1 | M-I, M-Z, I-Z | Gujarat, India | 21.50°N, 72.03°E | 579 | 27 | 0-20 | 7 years |
| 44 | Zhang, 2015[[44](#_ENREF_44)] | 1 | M-Z, M-I, I-Z | Jiangxi, China | 26.73°N, 115.05°E | 1470 | 18 | 0-20 | 14 years |
| 45 | Arif, 2016[[45](#_ENREF_45)] | 1 | M-Z, M-I, I-Z, MI-M, MI-I | Faisalabad, Pakistan | 31.4°N, 73.05°E | 200 | NM | 0-15 | 2 years |
| 46 | Batabyal, 2016[[46](#_ENREF_46)] | 1 | M-Z, M-I, I-Z, MI-M, MI-I | Bidhan Chandra, India | 23.0°N, 89.0°E | 1480 | 24.3 | 0-20 | 5 years |
| 47 | Bharali, 2016[[47](#_ENREF_47)] | 1 | I-Z | Assam, India | 26.68°N, 92.83°'E | NM | NM | 0-15 | 6 months |
| 48 | Li, 2016[[48](#_ENREF_48)] | 2 | MI-I, I-Z | Heilongjiang, China | 47.43°N, 126.63°E | 550 | 1.5 | 0-20 | 21 year |
| 49 | Mi, 2016[[49](#_ENREF_49)] | 3 | MI-I | Zhejiang, China | 29.17°N, 119.45°E | 1424 | 17.5 | 0-5  5-10  10-20 | 4 years |
| 50 | [Olatz](https://pubmed.ncbi.nlm.nih.gov/?term=Garaiyurrebaso+O&cauthor_id=28011599), 2016[[50](#_ENREF_50)] | 4 | M-Z | Basque Country, Spain | 43.22°N, 3.43°W | 1400 | 13 | NM | 40year |
| 51 | Qi, 2016[[51](#_ENREF_51)] | 1 | M-I, M-Z, I-Z | Shandong, China | 36.83°N, 116.57°E | 569 | 13.4 | 0-20 | 36 years |
| 52 | Qiu, 2016[[52](#_ENREF_52)] | 3 | MI-I | Jilin, China | 43.50°N, 124.80°E | 700 | 4.5 | 0-10  10-20  20-30 | 22 years |
| 53 | Singh, 2016[[53](#_ENREF_53)] | 1 | M-Z | Himachal Pradesh, India | 32.02°N, 76.70°E | 900 | 16.5 | 0-10 | 50 years |
| 54 | Kiani, 2017[[54](#_ENREF_54)] | 1 | M-Z, M-I, I-Z | Breton, Canada | 53.09°N, 114.44°W | 547 | NM | 0-5 | NM |
| 55 | Li, 2017[[55](#_ENREF_55)] | 1 | MI-I | Henan, China | 35.18°N, 113.68°E | 656 | 14 | 0-20 | 10 years |
| 56 | Liu, 2017[[56](#_ENREF_56)] | 2 | MI-I, I-Z  MI-M | Hubei, China | 30.85°N113.12°E | 1179 | 21 | 0-20 | 1 year  2 years |
| 57 | Freitas, 2018[[57](#_ENREF_57)] | 2 | I-Z | Catalonia, S[[8](#_ENREF_8)]pain | 41.55°N2.18°E | 647 | 14.9 | 0-10 | 4 years |
| 58 | Guo, 2018[[58](#_ENREF_58)] | 2 | I-Z，MI-M, MI-I | Anhui, China | 33.22°N, 116.62°E | 900 | 16.5 | 0-15 | 34 years |
| 59 | Guo, 2018[[59](#_ENREF_59)] | 1 | M-Z | Jiangsu, China | 31.45°N, 119.32°E | 1149.7 | 17.5 | 0-20 | 4 years |
| 60 | Hu, 2018[[60](#_ENREF_60)] | 3 | M-Z, M-I, I-Z, MI-M, MI-I | Heihe, China  Mingzhuxiang, China  Gongzhuling, China | 50.25°N, 127.45°E  45.83°N, 126◦83°E  43.85°N, 124.80°E | NM | NM | 0-20 | 3 years |
| 61 | Kiboi, 2018[[61](#_ENREF_61)] | 2 | M-Z, M-I, I-Z, MI-M, MI-I | Meru South, Kenya  Kandara, Kenya | 0.98°S, 37.13°E  0.33°S, 37.68°E | 1675  804 | 20.3  20.5 | 0-20 | 1 year |
| 62 | Li, 2018[[62](#_ENREF_62)] | 12 | M-Z | Hunan, China  Hunan, China  Hunan, China | 26.75°N, 111.87°E  35°N, 113.68°E  43.5°N, 124.8°E | 1255  575  575 | 18  14.5  4.5 | 10 | 1 month  12 months |
| 63 | Liu, 2018[[63](#_ENREF_63)] | 4 | M-I, M-Z, I-Z | Queensland, Australia | 25.55°S, 152.55°E | 1138 | 21.2 | 0-10  10-25  25-40  40-60 | 1 year |
| 64 | Newton, 2018[[64](#_ENREF_64)] | 60 | M-Z | Alberta, Canada | 50.05°N, 112.15°W | NM | 5.7 | 0-7.5 | 3-12 years |
| 65 | Pokhare, 2018[[65](#_ENREF_65)] | 2 | I-Z | Alberta, Canada | 53.18°N, 113.98°W | NM | NM | 0-10 | 1 year |
| 66 | Smitha, 2018[[66](#_ENREF_66)] | 1 | M-Z | Gujarat, India | 22.58°N, 72.92°E | 866 | NM | 0-15 | 2 years |
| 67 | Ali, 2019[[67](#_ENREF_67)] | 5 | I-Z | Islamabad, Pakistan | 33.68°N, 73.03°E | NM | NM | 0-30 | 1 year |
| 68 | Dubey, 2019[[68](#_ENREF_68)] | 2 | M-Z | Uttar Pradesh, India | 25.03°'N83°E | NM | NM | NM | 1 year |
| 69 | Hu, 2019[[69](#_ENREF_69)] | 4 | M-Z | Inner Mongolia, China | 46.28°N, 123.00°E | 375 | 4 | NM | NM |
| 70 | Lupwayi, 2019[[70](#_ENREF_70)] | 2 | M-Z, M-I, I-Z | Alberta, Canada | 49.7°N, 112.8°W | 250 | 14.7 | 0-15 | 30 years |
| 71 | Sato, 2019[[71](#_ENREF_71)] | 1 | M-I | Planaltina, DF, Brazil | 15.65°S, 47.73°W | 1400 | NM | 0-30 | NM |
| 72 | Ye, 2019[[72](#_ENREF_72)] | 2 | MI-I, I-Z  MI-M | Henan, China | 35.0°N, 113.68°E. | 645 | 14 | 0-7.5  7.5-20 | 26 years |
| 73 | Zhou, 2019[[73](#_ENREF_73)] | 1 | M-Z, M-I, I-Z, MI-M, MI-I | Guizhou, China | 26.45°N, 105.53°E | 1396.9 | 15.1 | 0-15 | 1year |
| 74 | Chen, 2020[[74](#_ENREF_74)] | 4 | M-I, M-Z, I-Z | Hunan, China | 28.02°N, 112.30°E | 1550 | 17.2 | 0-15 | NM |
| 75 | Ma, 2020[[75](#_ENREF_75)] | 4 | M-Z | SE England | 51.98°N, 36.93°W | NM | NM | 0-23  23-38 | 16 years  28 years |
| 76 | Samson, 2020[[76](#_ENREF_76)] | 6 | M-I, | Quebec City, Canada | 46.73°N, 71.52°W | NM | NM | 0-10 | 2 years |
| 77 | Xu, 2020[[77](#_ENREF_77)] | 2 | M-I, M-Z, I-Z | Hubei, China | 30.02°N, 114.35°E | 1300 | 16.8 | 0-20 | NM |
| 78 | Zhang, 2020[[78](#_ENREF_78)] | 2 | M-Z | Zhejiang, China | 29.28°N, 119.33°E | NM | 16.3 | 0-30 | NM |
| 79 | Zhu, 2020[[79](#_ENREF_79)] | 1 | M-Z, M-I, I-Z, MI-M, MI-I | Shandong, China | 36.83°N, 116.57°E | 710 | 12.4 | 0-30 | NM |

Depth refers to the depth range of the data extracted from the literature.

The minutes and seconds of longitude and latitude were converted to degrees.

NM, not mentioned.

Reference

1. Lundquist EJ, Jackson LE, Scow KM. Wet–dry cycles affect dissolved organic carbon in two California agricultural soils. Soil Biology and Biochemistry. 1999;31(7):1031-8. doi: <https://doi.org/10.1016/S0038-0717(99)00017-6>.

2. Kanchikerimath M, Singh D. Soil organic matter and biological properties after 26 years of maize–wheat–cowpea cropping as affected by manure and fertilization in a Cambisol in semiarid region of India. Agriculture, Ecosystems & Environment. 2001;86:155-62. doi: 10.1016/S0167-8809(00)00280-2.

3. Wu TY, Schoenau JJ, Li FM, Xu FL. Influence of cultivation and fertilization on total organic carbon and carbon fractions in soils from the Loess Plateau of China. Soil and Tillage Research. 2004;77(1):59-68. doi: 10.1016/j.still.2003.10.002. PMID: 004202874.

4. Manna MC, Swarup A, Wanjari RH, Ravankar HN, Mishra B, Saha MN, et al. Long-term effect of fertilizer and manure application on soil organic carbon storage, soil quality and yield sustainability under sub-humid and semi-arid tropical India. Field Crops Research. 2005;93(2):264-80. doi: <https://doi.org/10.1016/j.fcr.2004.10.006>.

5. Bhattacharyya P, Chakrabarti K, Chakraborty A. Microbial biomass and enzyme activities in submerged rice soil amended with municipal solid waste compost and decomposed cow manure. Chemosphere. 2005;60(3):310-8. doi: 10.1016/j.chemosphere.2004.11.097. PMID: 15924949.

6. Deng SP, Parham JA, Hattey J, Babu D. Animal manure and anhydrous ammonia amendment alter microbial carbon use efficiency, microbial biomass, and activities of dehydrogenase and amidohydrolases in semiarid agroecosystems. Applied Soil Ecology. 2006;33:258-68. doi: 10.1016/j.apsoil.2005.10.004.

7. Lupwayi NZ, Lea T, Beaudoin JL, Clayton GW. Soil microbial biomass, functional diversity and crop yields following application of cattle manure, hog manure and inorganic fertilizers. Canadian Journal of Soil Science. 2005;85:193-201.

8. Masto R, Chhonkar P, Singh D, Patra A. Changes in soil biological and biochemical characteristics in a long-term field trial on a sub-tropical inceptisol. Soil Biology and Biochemistry. 2006;38:1577-82. doi: 10.1016/j.soilbio.2005.11.012.

9. Pal R, Chakrabarti K, Chakraborty A, Chowdhury A. Pencycuron application to soils: degradation and effect on microbiological parameters. Chemosphere. 2005;60(11):1513-22. doi: 10.1016/j.chemosphere.2005.02.068. PMID: 16083758.

10. Rudrappa L, Purakayastha T, Singh D, Bhadraray S. Long-term manuring and fertilization effects on soil organic carbon pools in a Typic Haplustept of semi-arid sub-tropical India. Soil and Tillage Research. 2006;88:180-92. doi: 10.1016/j.still.2005.05.008.

11. Manna MC, Swarup A, Wanjari RH, Mishra B, Shahi DK. Long-term fertilization, manure and liming effects on soil organic matter and crop yields. Soil and Tillage Research. 2007;94(2):397-409. doi: <https://doi.org/10.1016/j.still.2006.08.013>.

12. Hernandez D, Fernandez JM, Plaza C, Polo A. Water-soluble organic matter and biological activity of a degraded soil amended with pig slurry. Science of the Total Environment. 2007;378(1-2):101-3. doi: 10.1016/j.scitotenv.2007.01.020. PMID: 17320153.

13. Pal R, Das P, Chakrabarti K, Chakraborty A, Chowdhury A. Side effects of pencycuron on nontarget soil microorganisms in waterlogged soil: Field experiment. Applied Soil Ecology. 2008;38(2):161-7. doi: 10.1016/j.apsoil.2007.10.005. PMID: IND43994263.

14. Challagulla V, Wani S, Ch S, Padmaja B, Vittal KPR. Microbial properties of soil as affected by cropping and nutrient management practices in several long term manurial experiments in the semi arid tropics of India. Applied Soil Ecology. 2008;40:165-73. doi: 10.1016/j.apsoil.2008.04.001.

15. Gu Y, Zhang X, Tu S, Lindström K. Soil microbial biomass, crop yields, and bacterial community structure as affected by long-term fertilizer treatments under wheat-rice cropping. European Journal of Soil Biology. 2009;45(3):239-46. doi: <https://doi.org/10.1016/j.ejsobi.2009.02.005>.

16. Liu E, Yan C, Mei X, He W, Bing SH, Ding L, et al. Long-term effect of chemical fertilizer, straw, and manure on soil chemical and biological properties in northwest China. Geoderma. 2010;158(3):173-80. doi: <https://doi.org/10.1016/j.geoderma.2010.04.029>.

17. Abbasi MK, Khizar A. Microbial biomass carbon and nitrogen transformations in a loam soil amended with organic–inorganic N sources and their effect on growth and N-uptake in maize. Ecological Engineering. 2012;39:123-32. doi: <https://doi.org/10.1016/j.ecoleng.2011.12.027>.

18. Li X, Han X, Li H, Song C, Yan J, Liang Y. Soil chemical and biological properties affected by 21-year application of composted manure with chemical fertilizers in a Chinese Mollisol. Canadian Journal of Soil Science. 2012;92:419-28. doi: 10.4141/cjss2010-046.

19. Lou Y, Wang J, Liang W. Impacts of 22-year organic and inorganic N managements on soil organic C fractions in a maize field, northeast China. Catena. 2011;87:386-90. doi: 10.1016/j.catena.2011.07.006.

20. Nayak AK, Gangwar B, Shukla AK, Mazumdar SP, Kumar A, Raja R, et al. Long-term effect of different integrated nutrient management on soil organic carbon and its fractions and sustainability of rice–wheat system in Indo Gangetic Plains of India. Field crops research. 2012;127:129-39. doi: 10.1016/j.fcr.2011.11.011. PMID: IND44675740.

21. Yu Z, Wang G, Jin J, Liu J, Liu X. Soil microbial communities are affected more by land use than seasonal variation in restored grassland and cultivated Mollisols in Northeast China. European Journal of Soil Biology. 2011;47(6):357-63. doi: <https://doi.org/10.1016/j.ejsobi.2011.09.001>.

22. Biau A, Santiveri F, Mijangos I, Lloveras J. The impact of organic and mineral fertilizers on soil quality parameters and the productivity of irrigated maize crops in semiarid regions. European Journal of Soil Biology. 2012;53:56–61. doi: 10.1016/j.ejsobi.2012.08.008.

23. Moharana P, Sharma BM, Biswas D, Dwivedi B, Singh RV. Long-term effect of nutrient management on soil fertility and soil organic carbon pools under a 6-year-old pearl millet-wheat cropping system in an Inceptisol of subtropical India. Field Crops Research. 2012;136:32–41. doi: 10.1016/j.fcr.2012.07.002.

24. Ghosh S, Wilson B, Ghoshal S, Senapati N, Mandal B. Organic amendments influence soil quality and carbon sequestration in the Indo-Gangetic plains of India. Agriculture, ecosystems and environment. 2012;156:134-41. doi: 10.1016/j.agee.2012.05.009. PMID: IND44715991.

25. Hurisso, Davis J, Brummer J, Stromberger M, Mikha M, Haddix M, et al. Rapid changes in microbial biomass and aggregate size distribution in response to changes in organic matter management in grass pasture. Geoderma. 2013;193-194:68-75. doi: 10.1016/j.geoderma.2012.10.016.

26. Poulsen P, Magid J, Luxhøi J, de Neergaard A. Effects of fertilization with urban and agricultural organic wastes in a field trial – Waste imprint on soil microbial activity. Soil Biology and Biochemistry. 2013;57:794–802. doi: 10.1016/j.soilbio.2012.02.031.

27. Ahmad W, Khan F, Shah PDZ, Jamal M, Ali K. Recovery of organic fertility in degraded soil through fertilization and crop rotation. Journal of the Saudi Society of Agricultural Sciences. 2013;13. doi: 10.1016/j.jssas.2013.01.007.

28. Said Al Busaidi K, Buerkert A, Joergensen R. Carbon and nitrogen mineralization at different salinity levels in Omani low organic matter soils. Journal of Arid Environments. 2014;s 100–101:106–10. doi: 10.1016/j.jaridenv.2013.10.013.

29. Plaza-Bonilla D, álvaro-Fuentes J, Cantero-Martínez CJSSSoAJ. Soil Aggregate Stability as Affected by Fertilization Type under Semiarid No-Tillage Conditions. Soil Science Society of America Journal. 2013;77(1):284. doi: 10.2136/sssaj2012.0258.

30. Das S, Adhya T. Effect of combine application of organic manure and inorganic fertilizer on methane and nitrous oxide emissions from a tropical flooded soil planted to rice. Geoderma. 2014;213:185-92. doi: 10.1016/j.geoderma.2013.08.011.

31. Mandal N, Brahma SD, Brij MS, Dhyan S, Mahesh CM, Rakesh KT, et al. Effect of induced defoliation in pigeonpea, farmyard manure and sulphitation pressmud on soil organic carbon fractions, mineral nitrogen and crop yields in a pigeonpea–wheat cropping system. Field crops research. 2013;154:178-87. doi: 10.1016/j.fcr.2013.08.007. PMID: IND500708541.

32. Manna M, Bhattacharyya P, Adhya T, Singh M, Wanjari R, Ramana S, et al. Carbon fractions and productivity under changed climate scenario in soybean–wheat system. Field Crops Research. 2013;145:10–20. doi: 10.1016/j.fcr.2013.02.004.

33. XU Y, LIU H, WANG X, XU M, ZHANG W, JIANG G. Changes in Organic Carbon Index of Grey Desert Soil in Northwest China After Long-Term Fertilization. Journal of Integrative Agriculture. 2014;13:554-61.

34. Plaza-Bonilla D, Cantero-Martínez C, Álvaro-Fuentes J. Soil management effects on greenhouse gases production at the macroaggregate scale. Soil Biology and Biochemistry. 2014;68:471-81. doi: 10.1016/j.soilbio.2013.10.026.

35. Balota EL, Machineski O, Hamid KI, Yada IF, Barbosa GM, Nakatani AS, et al. Soil microbial properties after long-term swine slurry application to conventional and no-tillage systems in Brazil. Science of the Total Environment. 2014;490:397-404. doi: 10.1016/j.scitotenv.2014.05.019. PMID: 24867704.

36. Luo P, Han X, Wang Y, Han M, Shi H, Liu N, et al. Influence of long-term fertilization on soil microbial biomass, dehydrogenase activity, and bacterial and fungal community structure in a brown soil of northeast China. Annals of microbiology. 2015;65(1):533-42. doi: 10.1007/s13213-014-0889-9. PMID: 25705148.

37. Zhang X, Wu X, Zhang S, Xing Y, Wang R, Liang W. Organic amendment effects on aggregate-associated organic C, microbial biomass C and glomalin in agricultural soils. Catena. 2014;123:188–94. doi: 10.1016/j.catena.2014.08.011.

38. Singh A, Singh MK, Ghoshal N. Microbial Biomass Dynamics in a Tropical Agroecosystem: Influence of Herbicide and Soil Amendments. Pedosphere. 2016;26(2):257-64. doi: <https://doi.org/10.1016/S1002-0160(15)60040-6>.

39. He YT, Zhang WJ, Xu MG, Tong XG, Sun FX, Wang JZ, et al. Long-term combined chemical and manure fertilizations increase soil organic carbon and total nitrogen in aggregate fractions at three typical cropland soils in China. Science of the Total Environment. 2015;532:635-44. doi: 10.1016/j.scitotenv.2015.06.011. PMID: 26119378.

40. Li J, Cooper JM, Lin Za, Li Y, Yang X, Zhao B. Soil microbial community structure and function are significantly affected by long-term organic and mineral fertilization regimes in the North China Plain. Applied Soil Ecology. 2015;96:75-87. doi: <https://doi.org/10.1016/j.apsoil.2015.07.001>.

41. Lu H, Lashari MS, Liu X, Ji H, Li L, Zheng J, et al. Changes in soil microbial community structure and enzyme activity with amendment of biochar-manure compost and pyroligneous solution in a saline soil from Central China. European Journal of Soil Biology. 2015;70:67-76. doi: <https://doi.org/10.1016/j.ejsobi.2015.07.005>.

42. Singh SR, Kundu DK, Tripathi MK, Dey P, Saha AR, Kumar M, et al. Impact of balanced fertilization on nutrient acquisition, fibre yield of jute and soil quality in New Gangetic alluvial soils of India. Applied Soil Ecology. 2015;92:24-34. doi: <https://doi.org/10.1016/j.apsoil.2015.03.007>.

43. Gopalakrishnan VA, Kubavat D, Trivedi K, Agarwal P, Wheeler C, Ghosh A. Long-term application of Jatropha press cake promotes seed yield by enhanced soil organic carbon accumulation, microbial biomass and enzymatic activities in soils of semi-arid tropical wastelands. European Journal of Soil Biology. 2015;69. doi: 10.1016/j.ejsobi.2015.05.005.

44. Zhang X, Dong W, Dai X, Schaeffer S, Yang F, Radosevich M, et al. Responses of absolute and specific soil enzyme activities to long term additions of organic and mineral fertilizer. Science of the Total Environment. 2015;536:59-67. doi: 10.1016/j.scitotenv.2015.07.043. PMID: 26196069.

45. Arif M, Riaz M, Shahzad S, Yasmeen T, Akhtar M, Riaz M, et al. Associative interplay of plant growth promoting rhizobacteria (Pseudomonas aeruginosa QS40) with nitrogen fertilizers improves sunflower (Helianthus annuus L.) productivity and fertility of aridisol. Applied Soil Ecology. 2016;108:238-47. doi: 10.1016/j.apsoil.2016.08.016.

46. Batabyal K, Mandal B, Sarkar D, Murmu S, Tamang A, Das I, et al. Comprehensive assessment of nutrient management technologies for cauliflower production under subtropical conditions. European Journal of Agronomy. 2016;79:1-13. doi: <https://doi.org/10.1016/j.eja.2016.04.009>.

47. Bharali A, Baruah K, Bhattacharyya P, Gorh D. Integrated nutrient management in wheat grown in a northeast India soil: Impacts on soil organic carbon fractions in relation to grain yield. Soil and Tillage Research. 2017;168:81-91. doi: 10.1016/j.still.2016.12.001.

48. Li L-J, Han X-Z. Changes of soil properties and carbon fractions after long-term application of organic amendments in Mollisols. Catena. 2016;143:140-4. doi: 10.1016/j.catena.2016.04.007.

49. Mi W, Wu L, Brookes P, Liu Y, Zhang X, Yang X. Changes in soil organic carbon fractions under integrated management systems in a low-productivity paddy soil given different organic amendments and chemical fertilizers. Soil and Tillage Research. 2016;163:64-70. doi: 10.1016/j.still.2016.05.009.

50. Garaiyurrebaso O, Garbisu C, Blanco F, Lanzén A, Martín I, Epelde L, et al. Long-term effects of aided phytostabilisation on microbial communities of metal-contaminated mine soil. FEMS microbiology ecology. 2017;93(3). doi: 10.1093/femsec/fiw252. PMID: 28011599.

51. Qi R, Li J, Lin Z, Li Z, Li Y, Yang X, et al. Temperature effects on soil organic carbon, soil labile organic carbon fractions, and soil enzyme activities under long-term fertilization regimes. Applied Soil Ecology. 2016;102:36-45. doi: 10.1016/j.apsoil.2016.02.004.

52. Qiu S, Gao H, Zhu P, Hou Y, Zhao S, Rong X, et al. Changes in soil carbon and nitrogen pools in a Mollisol after long-term fallow or application of chemical fertilizers, straw or manures. Soil and Tillage Research. 2016;163:255-65. doi: <https://doi.org/10.1016/j.still.2016.07.002>.

53. Singh P, Mitra S, Majumdar D, Bhattacharyya P, Prakash A, Borah P, et al. Nutrient and enzyme mobilization in earthworm casts: A comparative study with addition of selective amendments in undisturbed and agricultural soils of a mountain ecosystem. International Biodeterioration & Biodegradation. 2017;119:437-47. doi: <https://doi.org/10.1016/j.ibiod.2016.09.008>.

54. Kiani M, Hernandez-Ramirez G, Quideau S, Smith E, Janzen H, Larney F, et al. Quantifying sensitive soil quality indicators across contrasting long-term land management systems: Crop rotations and nutrient regimes. Agriculture, Ecosystems & Environment. 2017;248:123-35. doi: 10.1016/j.agee.2017.07.018.

55. Li C-x, Ma S-c, Shao Y, Ma S-T, Zhang L-l. Effects of long-term organic fertilization on soil microbiologic characteristics, yield and sustainable production of winter wheat. Journal of Integrative Agriculture. 2018;17:210-9. doi: 10.1016/S2095-3119(17)61740-4.

56. Liu Z, Rong Q, Zhou W, Liang G. Effects of inorganic and organic amendment on soil chemical properties, enzyme activities, microbial community and soil quality in yellow clayey soil. PLoS One. 2017;12:e0172767. doi: 10.1371/journal.pone.0172767.

57. Baldivieso-Freitas P, Blanco-Moreno JM, Armengot L, Chamorro L, Romanyà J, Sans FX. Crop yield, weed infestation and soil fertility responses to contrasted ploughing intensity and manure additions in a Mediterranean organic crop rotation. Soil and Tillage Research. 2018;180:10-20. doi: <https://doi.org/10.1016/j.still.2018.02.006>.

58. Guo Z, Zhang Z, Zhou H, Rahman MT, Wang DZ, Guo XS, et al. Long-term animal manure application promoted biological binding agents but not soil aggregation in a Vertisol. Soil and Tillage Research. 2018;180:232-7. doi: 10.1016/j.still.2018.03.007.

59. Guo J, Liu W, Zhu C, Luo G, Kong Y, Ling N, et al. Bacterial rather than fungal community composition is associated with microbial activities and nutrient-use efficiencies in a paddy soil with short-term organic amendments. Plant Soil. 2018;424:335-49.

60. Hu X, Liu J, Wei D, Zhu P, Cui Xa, Zhou B, et al. Soil Bacterial Communities Under Different Long-Term Fertilization Regimes in Three Locations Across the Black Soil Region of Northeast China. Pedosphere. 2018;28:751-63. doi: 10.1016/S1002-0160(18)60040-2.

61. Kiboi MN, Ngetich KF, Mugendi DN, Muriuki A, Adamtey N, Fliessbach A. Microbial biomass and acid phosphomonoesterase activity in soils of the Central Highlands of Kenya. Geoderma Regional. 2018;15:e00193. doi: <https://doi.org/10.1016/j.geodrs.2018.e00193>.

62. Li L, Xu M, Ali M, Zhang W, Duan Y, Li D. Factors affecting soil microbial biomass and functional diversity with the application of organic amendments in three contrasting cropland soils during a field experiment. PLoS One. 2018;13:e0203812. doi: 10.1371/journal.pone.0203812.

63. Liu X, Rashti MR, Dougall A, Esfandbod M, Zwieten LV, Chen C. Subsoil application of compost improved sugarcane yield through enhanced supply and cycling of soil labile organic carbon and nitrogen in an acidic soil at tropical Australia. Soil and Tillage Research. 2018;180:73-81.

64. Lupwayi NZ, Larney FJ, Blackshaw RE, Pearson DC, Eastman AH. Soil Microbial Biomass and Its Relationship With Yields of Irrigated Wheat Under Long-term Conservation Management. Soil Science. 2018;183(5):179-87. doi: 10.1097/ss.0000000000000242. PMID: 00010694-201809000-00002.

65. Pokharel P, Chang S. Manure pellet, woodchip and their biochars differently affect wheat yield and carbon dioxide emission from bulk and rhizosphere soils. Science of The Total Environment. 2018;659. doi: 10.1016/j.scitotenv.2018.12.380.

66. Gingade S, Basak B, Thondaiman V, Saha A. Nutrient management through organics, bio-fertilizers and crop residues improves growth, yield and quality of sacred basil (Ocimum sanctum Linn). Industrial Crops and Products. 2018;128:599-606. doi: 10.1016/j.indcrop.2018.11.058.

67. Ali B, Shah GA, Traore B, Shah S, Shah S-u-S, Al-Solaimani S, et al. Manure storage operations mitigate nutrient losses and their products can sustain soil fertility and enhance wheat productivity. Journal of Environmental Management. 2019;241. doi: 10.1016/j.jenvman.2019.02.081.

68. Dubey R, Dubey P, Abhilash P. Sustainable soil amendments for improving the soil quality, yield and nutrient content of Brassica juncea (L.) grown in different agroecological zones of eastern Uttar Pradesh, India. Soil and Tillage Research. 2019;195. doi: 10.1016/j.still.2019.104418.

69. Hu J, Wu J, Qu X. Effects of Organic Wastes on Labile Organic Carbon in Semiarid Soil under Plastic Mulched Drip Irrigation. Archives of Agronomy and Soil Science. 2019;65(13):1873-84.

70. Lupwayi N, Zhang Y, Hao X, Thomas B, Eastman A, Schwinghamer T. Linking soil microbial biomass and enzyme activities to long-term manure applications and their nonlinear legacy. Pedobiologia. 2019;74. doi: 10.1016/j.pedobi.2019.04.001.

71. Sato JH, Figueiredo CCd, Marchão RL, Oliveira ADd, Vilela L, Delvico FM, et al. Understanding the relations between soil organic matter fractions and N_2_O emissions in a long-term integrated crop–livestock system. European Journal of Soil Science. 2019:1-14.

72. Ye H, Lu C, Lin Q. Investigation of the spatial heterogeneity of soil microbial biomass carbon and nitrogen under long-term fertilizations in fluvo-aquic soil. PLoS One. 2019;14(4):e0209635. doi: 10.1371/journal.pone.0209635. PMID: 30947266.

73. Zhou Z, Gao T, Zhu Q, Yan T, Li D, Xue J, et al. Increases in bacterial community network complexity induced by biochar-based fertilizer amendments to karst calcareous soil. Geoderma. 2019;337:691-700. doi: <https://doi.org/10.1016/j.geoderma.2018.10.013>.

74. Chen X, Xia Y, Rui Y, Ning Z, Hu Y, Tang H, et al. Microbial carbon use efficiency, biomass turnover, and necromass accumulation in paddy soil depending on fertilization. Agriculture, Ecosystems & Environment. 2020;292:106816. doi: <https://doi.org/10.1016/j.agee.2020.106816>.

75. Ma Q, Wen Y, Wang D, Sun X, Hill PW, Macdonald A, et al. Farmyard manure applications stimulate soil carbon and nitrogen cycling by boosting microbial biomass rather than changing its community composition. Soil Biology and Biochemistry. 2020;144:107760. doi: <https://doi.org/10.1016/j.soilbio.2020.107760>.

76. Samson M-E, Chantigny MH, Vanasse A, Menasseri-Aubry S, Royer I, Angers DA. Management practices differently affect particulate and mineral-associated organic matter and their precursors in arable soils. Soil Biology and Biochemistry. 2020;148:107867. doi: <https://doi.org/10.1016/j.soilbio.2020.107867>.

77. Xu P, Liu Y, Zhu J, Shi L, Fu Q, Chen J, et al. Influence mechanisms of long-term fertilizations on the mineralization of organic matter in Ultisol. Soil and Tillage Research. 2020;201:104594. doi: 10.1016/j.still.2020.104594.

78. Zhang K, Chen L, Li Y, Brookes PC, Xu J, Luo Y. Interactive effects of soil pH and substrate quality on microbial utilization. European Journal of Soil Biology. 2020;96:103151. doi: <https://doi.org/10.1016/j.ejsobi.2020.103151>.

79. Zhu Z, Bai Y, Lv M, Tian G, Zhang X, Li L, et al. Soil Fertility, Microbial Biomass, and Microbial Functional Diversity Responses to Four Years Fertilization in an Apple Orchard in North China. Horticultural Plant Journal. 2020. doi: <https://doi.org/10.1016/j.hpj.2020.06.003>.
