# Supplementary material for: A global meta-analysis of animal manure application and soil microbial ecology based on random control treatments
Source: PLoS One. 2022 Jan 21;17(1):e0262139. doi: 10.1371/journal.pone.0262139 (PMC8782357; doi:10.1371/journal.pone.0262139)
Supplement: S2 Table — (DOCX) [file pone.0262139.s005.docx]

Table S2. Characteristics of the Shannon index studies included in the meta-analysis.

| No. | Study ID | Data set  No. | Dataset group | Location | Latitude and longitude | Annual  precipitation  (mm) | Annual average  temperature (℃) | Depth (cm) | Duration |
| --- | --- | --- | --- | --- | --- | --- | --- | --- | --- |
| 1 | Lupwayi, 2005[[1](#_ENREF_1)] | 14 | M-Z, M-I, I-Z | Falher Alberta, Canada  Fairview Alberta, Canada | 55.72°N, 117.20°W  56.06°N, 118.38°W | 367 | NM | 0-15 | 1-3 years |
| 2 | Mijangos, 2005[[2](#_ENREF_2)] | 1 | M-Z, M-I, I-Z | Basque, Spain | 42.52°N, 2.43°W | 1200 | 13.5 | 0-10 | 12 years |
| 3 | Nahar, 2006[[3](#_ENREF_3)] | 8 | M-Z | Wooster, Ohio, USA | 40.80°N, 81.93°W | NM | NM | 0-15 | 2 years |
| 4 | Okada, 2006[[4](#_ENREF_4)] | 2 | M-Z, M-I, I-Z | Fukushima Prefecture, Japan | 37.7°N, 140.38°E | 787.8 | 12.1 | 0-15 | 2 years |
| 5 | Ros, 2006[[5](#_ENREF_5)] | 1 | M-Z, M-I, I-Z, MI-M, MI-I | Linz, Austria | 48.3°N, 14.28°E | NM | NM | 0-20 | NM |
| 6 | Elgayar, 2009[[6](#_ENREF_6)] | 1 | M-I | Gharbia Governorate, Egypt | 30.6°N, 30.8°E | NM | NM | NM | 1 year |
| 7 | Gu, 2009[[7](#_ENREF_7)] | 2 | M-Z, M-I, I-Z, MI-M, MI-I | Sichuan, China | 30.17°N, 105.05°E | 1014 | 17.5 | 0-20 | 1 year |
| 8 | Ngosong, 2009[[8](#_ENREF_8)] | 4 | M-I | Darmstadt, Germany | 49°N,8°E | 590 | 9.5 | 0-5 | 1 year |
| 9 | Kamaa, 2011[[9](#_ENREF_9)] | 1 | M-Z, M-I, I-Z, MI-M, MI-I | Kabete, Kenya | 1.25°S, 36.68°E | 980 | 18.2 | 0-10 | 1 year |
| 10 | Dai, 2012[[10](#_ENREF_10)] | 1 | M-Z, M-I, I-Z | Henan, China | 35.07°N, 113.17°E | NM | NM | 0-15 | 2 year |
| 11 | Poulsen, 2012[[11](#_ENREF_11)] | 5 | M-I, M-Z, I-Z | Copenhagen, Denmark | 55.67°N, 12.30°E | NM | NM | 0-20 | 1 year |
| 12 | Rames, 2013[[12](#_ENREF_12)] | 3 | M-Z | Nambour QLD, Australia | 27.30°S, 153°E | 2500 | 20 |  | 3 years |
| 13 | Chen, 2014[[13](#_ENREF_13)] | 6 | M-Z, M-I, I-Z, MI-M, MI-I | Jiangxi, China | 28.25°N, 116.92°E | 1795 | 17.6 | 0-15 | 2 years |
| 14 | Luo, 2014[[14](#_ENREF_14)] | 1 | M-Z, M-I, I-Z, MI-M, MI-I | Liaoning , China | 40.8°N, 123.55°E | 629 | 7.5 | 0-20 | 32 years |
| 15 | Qiu, 2014[[15](#_ENREF_15)] | 1 | M-Z, M-I, I-Z, MI-M, MI-I | Fujian, China | 26.68°-27.4°N, 119.38°-119.85°E | 1540 | 19.3 | 0-20 | 5 years |
| 16 | Zhen, 2014[[16](#_ENREF_16)] | 3 | M-Z, M-I, I-Z | Shandong, China | 35.43°N, 117.83°E | 770.2 | 13.2 | 0-20 | NM |
| 17 | Calleja‐Cervantes, 2015[[17](#_ENREF_17)] | 2 | M-Z, M-I, I-Z | Navarra, Spain | 42.82°N, 1.64°W | 442 | 13..8 | 0-30 | 12 years |
| 18 | Lu, 2015[[18](#_ENREF_18)] | 4 | M-Z | Henan, China | 34.53°N, 115.50°E | 777 | 13.9 | 0-10 | 2 years |
| 19 | Li, 2015[[19](#_ENREF_19)] | 1 | M-Z, M-I, I-Z, MI-M, MI-I | Shandong, China | 36.83°N, 116.57°E | 569 | 13.4 | 0-20 | 15 years |
| 20 | Sale, 2015[[20](#_ENREF_20)] | 20 | M-Z, M-I, I-Z | Canton Aargau, Switzerland | 47.5°N, 8.02°E | 1000 | 9 | 0-40 | 4 years |
| 21 | Tian, 2015[[21](#_ENREF_21)] | 3 | M-Z | Jiangsu, China | 32.47°N, 119.1°W | NM | NM | 0-12 | 12 years |
| 22 | Wang, 2015[[22](#_ENREF_22)] | 1 | M-Z, M-I, I-Z, MI-M, MI-I | Shanxi, China | 35.2°N, 107.67°E | 584 | 9.1 | 0-20 | 30 years |
| 23 | Gai, 2016[[23](#_ENREF_23)] | 1 | M-Z, M-I, I-Z, MI-M, MI-I | Jiangsu, China | 31.5°N,120.1°E | 1086 | 15.7 | 0-20 | 5 years |
| 24 | Menezes-Blackburn, 2016[[24](#_ENREF_24)] | 3 | M-Z | Rio Blanco, Chile | 40.33°S, 72.58°W | NM | NM | NM | NM |
| 25 | Suleiman, 2016[[25](#_ENREF_25)] | 6 | M-Z, M-I, I-Z, MI-M, MI-I | Rio Grande do Sul State, Brazil | 29.72°S, 53.72°W | 1700 | 19.9 | 0-10 | 0-50 days |
| 26 | Ndeye, 2016[[26](#_ENREF_26)] | 4 | M-Z, M-I, I-Z, MI-M, MI-I | Burkina Faso | 12.27°N, 2.15°W | 800 | NM | 0-10 | 26 years |
| 27 | Das, 2017[[27](#_ENREF_27)] | 2 | M-Z | Gyeongsang, Korea | 35.1°N, 128.12°E | NM | NM | 0-20 | 1 year |
| 28 | Li, 2017[[28](#_ENREF_28)] | 5 | M-Z | Shandong, China | 37.8°N, 117.72°E | NM | NM | 0-20 | 13 months |
| 29 | Li, 2017[[29](#_ENREF_29)] | 1 | M-Z, M-I, I-Z, MI-M, MI-I | Hunan, China | 26.75°N, 111.87°E | NM | NM | 0-20 | NM |
| 30 | Jones, 2017[[30](#_ENREF_30)] | 1 | M-Z | Arkansas, USA | 36°N, 94°W | 1269 | 14 | 0-10 | 7 years |
| 31 | Peruzzi, 2017[[31](#_ENREF_31)] | 2 | M-Z, M-I, I-Z | South Tyrol, Italy | 46.62°N, 1.87°E | NM | NM | 0-30 | 5 years |
| 32 | Zhang, 2017[[32](#_ENREF_32)] | 9 | M-Z, M-I, I-Z | Alberta, Canada | 49.7°N, 112.8°W | 251 | 14.7 | NM | 30 years |
| 33 | Wang, 2017[[33](#_ENREF_33)] | 1 | I-Z, MI-I | Fujian, China | 26.68°-27.4°N, 119.38°-119.85°E | 1540 | 19.3 | 0-20 | 10 years |
| 34 | Cui, 2018[[34](#_ENREF_34)] | 1 | M-Z, M-I, I-Z, MI-M, MI-I | Hunan, China | 26.75°N, 111.87°E | NM | NM | 0-20 | NM |
| 35 | Han, 2018[[35](#_ENREF_35)] | 2 | M-Z, M-I, I-Z | Hunan, China | 26.75°N, 111.87°E | 1300 | 18 | 0-20 | 5 years |
| 36 | Ji, 2018[[36](#_ENREF_36)] | 3 | M-Z, M-I, I-Z | Fujian, China | 27.23°N, 119.57°E | 1646 | 19.3 | 0-10 | 1 year |
| 37 | Jia, 2018[[37](#_ENREF_37)] | 1 | M-Z | Shanxi, China | 37.97°N, 112.91°E | 450 | 9.5 | NM | 50 days |
| 38 | Li, 2018[[38](#_ENREF_38)] | 12 | M-Z | Hunan, China | 26.75°N, 111.87°E | 1255 | 18 | 10 | 1 -12 months |
| 39 | Manjunath, 2018[[39](#_ENREF_39)] | 1 | M-Z, M-I, I-Z | Varanasi, India | 25.10°N, 82.52°E | 1000 | 24.3 | 15 | NM |
| 40 | Tang, 2018[[40](#_ENREF_40)] | 2 | I-Z, MI-I | Hunan, China | 28.12°N, 112.3°E | 1354 | 17.2 | 0-20 | 3 years |
| 41 | Chen, 2019[[41](#_ENREF_41)] | 1 | I-Z, MI-I | Anhui, China | 33.62°N, 116.75°E | NM | NM | 0-20 | NM |
| 42 | Li, 2019[[42](#_ENREF_42)] | 8 | M-Z, M-I, I-Z | Saskatchewan, Canada | 50.28°N,107.8°W | NM | NM |  | 2-3 years |
| 43 | Tong, 2019[[43](#_ENREF_43)] | 3 | MI-I | Hebei, China | 36.9°N, 115.01°E | 556.2 | 13.1 | 0-20 | 16 years |
| 44 | Wang, 2019[[44](#_ENREF_44)] | 1 | I-Z, MI-I | Tengger Desert, China | 37.53°N, 105.03°E | <200 | 9.6 | 20 | 17 years |
| 45 | Yin, 2019[[45](#_ENREF_45)] | 1 | M-Z, M-I, I-Z, MI-M, MI-I | Xinjiang, China | 37.02°N, 80.72°E | 42 | 12.7 | 0-20 | 1 year |
| 46 | Zhu, 2019[[46](#_ENREF_46)] | 1 | M-Z | Zhejiang, China | 29.8°N, 121.38°E; | NM | NM | 0-20 | NM |
| 47 | Zhou, 2019[[47](#_ENREF_47)] | 1 | M-Z, M-I, I-Z, MI-M, MI-I | Guizhou, China | 26.45°N, 105.53°E | 1396.9 | 15.1 | 0-15 | 1 year |
| 48 | Escobar, 2020[[48](#_ENREF_48)] | 1 | M-I, MI-M, MI-I | Cundinamarca, Colombia | 4.33°N, 74.37°W | 1720 | 25 | NM | NM |
| 49 | Gryta, 2020[[49](#_ENREF_49)] | 1 | M-Z | Lower Silesia, Poland | 49.9°N, 19.2°E | NM | NM | 0-30 | NM |
| 50 | Huang, 2020[[50](#_ENREF_50)] | 3 | I-Z, MI-I | Chongqing, China | 30.43°N, 106.43°E | NM | NM | 0-20 | 1 year |
| 51 | Huang, 2020[[51](#_ENREF_51)] | 1 | I-Z, MI-I | Chongqing, China | 29.8°N, 106.4°E | 1105.5 | 18.4 | 0-20 | NM |
| 52 | Qin, 2020[[52](#_ENREF_52)] | 1 | I-Z, MI-I | Hebei, China | 36.87°N, 115.02°E | 494 | 13.2 | 0-20 | 10 years |
| 53 | Tao, 2020[[53](#_ENREF_53)] | 2 | M-Z, M-I, I-Z | Xinjiang, China | 44.38°N, 85.68°E | 225 | 7.8 | 0-20 | 3 years |
| 54 | Xu, 2020[[54](#_ENREF_54)] | 2 | M-Z, M-I, I-Z | Hubei, China | 30.02°N, 114.35°E | 1300 | 16.8 | 0-20 | NM |
| 55 | Zhu, 2020[[55](#_ENREF_55)] | 1 | M-Z, M-I, I-Z, MI-M, MI-I | Shandong, China | 36.83°N, 116.57°E | 710 | 12.4 | 0-30 | NM |

Depth refers to the depth range of the data extracted from the literature.

The minutes and seconds of longitude and latitude were converted to degrees.

NM, not mentioned.

Reference

1. Lupwayi NZ, Lea T, Beaudoin JL, Clayton GW. Soil microbial biomass, functional diversity and crop yields following application of cattle manure, hog manure and inorganic fertilizers. Canadian Journal of Soil Science. 2005;85:193-201. doi: 10.4141/s04-044

2. Mijangos I, Pérez R, Albizu I, Garbisu CJE, Technology M. Effects of fertilization and tillage on soil biological parameters. Enzyme and Microbial Technology. 2007;40(1):100-6. doi: 10.1016/j.enzmictec.2005.10.043

3. Nahar MS, Grewal PS, Miller SA, Stinner D, Stinner BR, Kleinhenz MD, et al. Differential effects of raw and composted manure on nematode community, and its indicative value for soil microbial, physical and chemical properties. Applied Soil Ecology. 2006;34(2-3):140-51. doi: 10.1016/j.apsoil.2006.03.011. PMID: WOS:000241084600005.

4. Okada H, Harada H. Effects of tillage and fertilizer on nematode communities in a Japanese soybean field. Applied Soil Ecology. 2007;35(3):582-98. doi: 10.1016/j.apsoil.2006.09.008. PMID: WOS:000244172100013.

5. Ros M, Klammer S, Knapp B, Aichberger K, Insam H. Long-term effects of compost amendment of soil on functional and structural diversity and microbial activity. Soil Use Manage. 2006;22(2):209-18. doi: 10.1111/j.1475-2743.2006.00027.x. PMID: WOS:000237516200012.

6. Elgayar EA, Alassiuty ANI, Alkorashy Mmjejoeb. Abundance and Species Diversity of Soil Microarthropods as Influenced by Different Types of Fertilizers in Greenhouse Biotopes. Journal of Experimental Biology. 2009; 5: 67– 75.

7. Gu Y, Zhang X, Tu S, Lindström K. Soil microbial biomass, crop yields, and bacterial community structure as affected by long-term fertilizer treatments under wheat-rice cropping. European Journal of Soil Biology. 2009;45(3):239-46. doi: <https://doi.org/10.1016/j.ejsobi.2009.02.005>.

8. Ngosong C, Raupp J, Scheu S, Ruess L. Low importance for a fungal based food web in arable soils under mineral and organic fertilization indicated by Collembola grazers. Soil Biology and Biochemistry. 2009;41(11):2308-17. doi: 10.1016/j.soilbio.2009.08.015. PMID: WOS:000271545500007.

9. Kamaa M, Mburu H, Blanchart E, Chibole L, Chotte JL, Kibunja C, et al. Effects of organic and inorganic fertilization on soil bacterial and fungal microbial diversity in the Kabete long-term trial, Kenya. Biology and Fertility of Soils. 2011;47(3):315-21. doi: 10.1007/s00374-011-0539-3. PMID: WOS:000288251700008.

10. Dai J, Hu J, Lin X, Yang A, Wang R, Zhang J, et al. Arbuscular mycorrhizal fungal diversity, external mycelium length, and glomalin-related soil protein content in response to long-term fertilizer management. Journal of Soils and Sediments. 2013;13(1):1-11.doi: 10.1007/s11368-012-0576-z

11. Poulsen P, Magid J, Luxhøi J, de Neergaard A. Effects of fertilization with urban and agricultural organic wastes in a field trial – Waste imprint on soil microbial activity. Soil Biology and Biochemistry. 2013;57:794–802. doi: 10.1016/j.soilbio.2012.02.031.

12. Rames EK, Smith MK, Hamill SD, De Faveri J. Microbial indicators related to yield and disease and changes in soil microbial community structure with ginger farm management practices.Australasian Plant Pathology. 2013;42(6):685-92. doi: 10.1007/s13313-013-0231-1. PMID: WOS:000325939100008.

13. Chen X, Li Z, Liu M, Jiang C, Che YJJoS, Sediments. Microbial community and functional diversity associated with different aggregate fractions of a paddy soil fertilized with organic manure and/or NPK fertilizer for 20years. Journal of Soils and Sediments. 2015;15(2):292-301. doi: 10.1007/s11368-014-0981-6

14. Luo PY, Han XR, Wang Y, Han M, Shi H, Liu N, et al. Influence of long-term fertilization on soil microbial biomass, dehydrogenase activity, and bacterial and fungal community structure in a brown soil of northeast China. Annals of Microbiology. 2015;65(1):533-42. doi: 10.1007/s13213-014-0889-9. PMID: WOS:000350219500052.

15. Qiu SL, Wang LM, Huang DF, Lin XJ. Effects of fertilization regimes on tea yields, soil fertility, and soil microbial diversity. Chilean Journal of Agricultural Research. 2014;74(3):333-9. doi: 10.4067/s0718-58392014000300012. PMID: WOS:000341345900012.

16. Zhen Z, Liu HT, Wang N, Guo LY, Meng J, Ding N, et al. Effects of Manure Compost Application on Soil Microbial Community Diversity and Soil Microenvironments in a Temperate Cropland in China. PLoS One. 2014;9(10). doi: 10.1371/journal.pone.0108555. PMID: WOS:000343730400016.

17. Calleja‐Cervantes ME. Changes in soil nutrient content and bacterial community after 12 years of organic amendment application to a vineyard. European journal of soil science. 2015;v. 66(no. 4):pp. 802-12-2015 v.66 no.4. doi: 10.1111/ejss.12261. PMID: 3427927.

18. Lu H, Lashari MS, Liu X, Ji H, Li L, Zheng J, et al. Changes in soil microbial community structure and enzyme activity with amendment of biochar-manure compost and pyroligneous solution in a saline soil from Central China. European Journal of Soil Biology. 2015;70:67-76. doi: <https://doi.org/10.1016/j.ejsobi.2015.07.005>.

19. Li J, Cooper JM, Lin ZA, Li YT, Yang XD, Zhao BQ. Soil microbial community structure and function are significantly affected by long-term organic and mineral fertilization regimes in the North China Plain. Applied Soil Ecology. 2015;96:75-87. doi: 10.1016/j.apsoil.2015.07.001. PMID: WOS:000361880400010.

20. Sale V, Aguilera P, Laczko E, Mader P, Berner A, Zihlmann U, et al. Impact of conservation tillage and organic farming on the diversity of arbuscular mycorrhizal fungi. Soil Biology and Biochemistry. 2015;84:38-52. doi: 10.1016/j.soilbio.2015.02.005. PMID: WOS:000353087600005.

21. Tian W, Zhang Z, Hu X, Tian R, Zhang J, Xiao X, et al. Short-term changes in total heavy metal concentration and bacterial community composition after replicated and heavy application of pig manure-based compost in an organic vegetable production system. Biology and Fertility of Soils. 2015;51(5):593-603. doi: 10.1007/s00374-015-1005-4.

22. Wang Y, Ji HF, Gao CQ. Differential responses of soil bacterial taxa to long-term P, N, and organic manure application. Journal of Soils and Sediments. 2016;16(3):1046-58. doi: 10.1007/s11368-015-1320-2. PMID: WOS:000370958100029.

23. Gai XP, Liu HB, Zhai LM, Tan GC, Liu J, Ren TZ, et al. Vegetable yields and soil biochemical properties as influenced by fertilization in Southern China. Applied Soil Ecology. 2016;107:170-81. doi: 10.1016/j.apsoil.2016.06.001. PMID: WOS:000384860400018.

24. Menezes-Blackburn D, Inostroza NG, Gianfreda L, Greiner R, Mora ML, Jorquera MA. Phytase-producing Bacillus sp inoculation increases phosphorus availability in cattle manure. Journal of Soil Science and Plant Nutrition. 2016;16(1):200-10. PMID: WOS:000377936900016.

25. Suleiman AKA, Gonzatto R, Aita C, Lupatini M, Jacques RJS, Kuramae EE, et al. Temporal variability of soil microbial communities after application of dicyandiamide-treated swine slurry and mineral fertilizers. Soil Biology and Biochemistry. 2016;97:71-82. doi: 10.1016/j.soilbio.2016.03.002. PMID: WOS:000375517400008.

26. Ndeye Hélène Diallo-Diagne, Assigbetse K, Sall S, Masse D, Bonzi M, Ndoye I, et al. Response of Soil Microbial Properties to Long-Term Application of Organic and Inorganic Amendments in a Tropical Soil (Saria, Burkina Faso). Open Journal of Soil Science,. 2016:21-33. doi: <http://dx.doi.org/10.4236/ojss.2016.62003>.

27. Das S, Jeong ST, Das S, Kim PJ. Composted Cattle Manure Increases Microbial Activity and Soil Fertility More Than Composted Swine Manure in a Submerged Rice Paddy. Frontiers in Microbiology. 2017;8:10. doi: 10.3389/fmicb.2017.01702. PMID: WOS:000409347100001.

28. Li GY, Wu CF. Effects of Short-Term Set-Aside Management Practices on Soil Microorganism and Enzyme Activity in China. International Journal of Environmental Research and Public Health. 2017;14(8):14. doi: 10.3390/ijerph14080913. PMID: WOS:000408684300083.

29. Li LH, Fan FL, Song AL, Yin C, Cui PY, Li ZJ, et al. Microbial composition and diversity are associated with plant performance: a case study on long-term fertilization effect on wheat growth in an Ultisol. Applied Microbiology and Biotechnology. 2017;101(11):4669-81. doi: 10.1007/s00253-017-8147-2. PMID: WOS:000402008300027.

30. Jones J, Savin MC, Rom CR, Gbur E. Denitrifier community response to seven years of ground cover and nutrient management in an organic fruit tree orchard soil. Applied Soil Ecology. 2017;112:60-70. doi: 10.1016/j.apsoil.2016.12.009. PMID: WOS:000393630900008.

31. Peruzzi E, Franke-Whittle IH, Kelderer M, Ciavatta C, Insam H. Microbial indication of soil health in apple orchards affected by replant disease. Applied Soil Ecology. 2017;119:115-27. doi: 10.1016/j.apsoil.2017.06.003. PMID: WOS:000408882500014.

32. Zhang YT, Hao XY, Alexander TW, Thomas B, Shi XJ, Lupwayi NZ. Long-term and legacy effects of manure application on soil microbial community composition. Biology and Fertility of Soils. 2018;54(2):269-83. doi: 10.1007/s00374-017-1257-2. PMID: WOS:000422837100008.

33. Wang LM, Huang DF, Fang Y, Wang F, Li FL, Liao M. Soil fungal communities in tea plantation after 10 years of chemical vs. integrated fertilization. Chilean Journal of Agricultural Research. 2017;77(4):355-64. doi: 10.4067/s0718-58392017000400355. PMID: WOS:000416141300009.

34. Cui XW, Zhang YZ, Gao JS, Peng FY, Gao P. Long-term combined application of manure and chemical fertilizer sustained higher nutrient status and rhizospheric bacterial diversity in reddish paddy soil of Central South China. Scientific Reports. 2018;8:11. doi: 10.1038/s41598-018-34685-0. PMID: WOS:000449499500043.

35. Han S, Zeng LY, Luo XS, Xiong X, Wen SL, Wang BR, et al. Shifts in Nitrobacter- and Nitrospira-like nitrite-oxidizing bacterial communities under long-term fertilization practices. Soil Biology and Biochemistry. 2018;124:118-25. doi: 10.1016/j.soilbio.2018.05.033. PMID: WOS:000444358200014.

36. Ji L, Wu Z, You Z, Yi X, Ni K, Guo S, et al. Effects of organic substitution for synthetic N fertilizer on soil bacterial diversity and community composition: A 10-year field trial in a tea plantation. Agriculture, Ecosystems & Environment. 2018;268:124-32. doi: 10.1016/j.agee.2018.09.008.

37. Jia Z, Jiang L, Hong L, Jing H, Zhe C, Yuanjun N, et al. Manipulation of the rhizosphere microbial community through application of a new bio-organic fertilizer improves watermelon quality and health. PLoS One 2018;13(2):e0192967. doi: 10.1371/journal.pone.0192967.

38. Li L, Xu MG, Ali ME, Zhang WJ, Duan YH, Li DC. Factors affecting soil microbial biomass and functional diversity with the application of organic amendments in three contrasting cropland soils during a field experiment. PloS One. 2018;13(9). doi: 10.1371/journal.pone.0203812. PMID: WOS:000444545800079.

39. Manjunath M, Kumar U, Yadava RB, Rai AB, Singh B. Influence of organic and inorganic sources of nutrients on the functional diversity of microbial communities in the vegetable cropping system of the Indo-Gangetic plains. Comptes Rendus Biologies. 2018;341(6):349-57. doi: 10.1016/j.crvi.2018.05.002. PMID: 29861196.

40. Tang HM, Xu YL, Xiao XP, Li C, Li WY, Cheng KK, et al. Impacts of long-term fertilization on the soil microbial communities in double-cropped paddy fields. Journal of Agricultural Science. 2018;156(7):857-64. doi: 10.1017/s0021859618000825. PMID: WOS:000454415900001.

41. Chen L, Li F, Li W, Ning Q, Li JW, Zhang JB, et al. Organic amendment mitigates the negative impacts of mineral fertilization on bacterial communities in Shajiang black soil. Applied Soil Ecology. 2020;150:7. doi: 10.1016/j.apsoil.2019.103457. PMID: WOS:000512886800008.

42. Li YL, Gan YT, Lupwayi N, Hamel C. Influence of introduced arbuscular mycorrhizal fungi and phosphorus sources on plant traits, soil properties, and rhizosphere microbial communities in organic legume-flax rotation. Plant Soil. 2019;443(1-2):87-106. doi: 10.1007/s11104-019-04213-8. PMID: WOS:000493661800006.

43. Tong LH, Zhu L, Lv YH, Zhu K, Liu XY, Zhao R. Response of organic carbon fractions and microbial community composition of soil aggregates to long-term fertilizations in an intensive greenhouse system. Journal of Soils and Sediments. 2020;20(2):641-52. doi: 10.1007/s11368-019-02436-x. PMID: WOS:000513281900006.

44. Wang ZR, Liu YB, Zhao LN, Zhang WL, Liu LC. Change of soil microbial community under long-term fertilization in a reclaimed sandy agricultural ecosystem. PeerJ. 2019;7:21. doi: 10.7717/peerj.6497. PMID: WOS:000459790200007.

45. Yin MY, Gao XP, Tenuta M, Kuang WN, Gui DW, Zeng FJ. Manure application increased denitrifying gene abundance in a drip-irrigated cotton field. PeerJ. 2019;7:21. doi: 10.7717/peerj.7894. PMID: WOS:000491858000005.

46. Zhu D, Xinag Q, Yang XR, Ke X, O'Connor P, Zhu YG. Trophic Transfer of Antibiotic Resistance Genes in a Soil Detritus Food Chain. Environmental Science & Technology. 2019;53(13):7770-81. doi: 10.1021/acs.est.9b00214. PMID: WOS:000474478300059.

47. Zhou Z, Gao T, Zhu Q, Yan T, Li D, Xue J, et al. Increases in bacterial community network complexity induced by biocharbased fertilizer amendments to karst calcareous soil. Geoderma. 2019;337:691-700. doi: <https://doi.org/10.1016/j.geoderma.2018.10.013>.

48. Escobar Escobar N, Arenas Suarez N, Marquez S. Characterization of microbial populations associated with different organic fertilizers. International Journal of Recycling of Organic Waste in Agriculture. 2020;9:171-82. doi: 10.30486/IJROWA.2020.1890242.1022.

49. Gryta A, Frąc M, Oszust K. Genetic and Metabolic Diversity of Soil Microbiome in Response to Exogenous Organic Matter Amendments. Agronomy. 2020, 10(4); 546; doi: 10.3390/agronomy10040546.

50. Huang R, Wang Y, Gao X, Liu J, Wang Z, Gao M. Nitrous oxide emission and the related denitrifier community: A short-termresponse to organic manure substituting chemical fertilizer. Ecotoxicology and Environmental Safety. 2020;192. doi: <https://doi.org/10.1016/j.ecoenv.2020.110291>.

51. Huang R, Wang YY, Liu J, Gao JJ, Zhang YR, Ni JP, et al. Partial substitution of chemical fertilizer by organic materials changed the abundance, diversity, and activity of nirS-type denitrifying bacterial communities in a vegetable soil. Applied Soil Ecology. 2020;152:9. doi: 10.1016/j.apsoil.2020.103589. PMID: WOS:000529336600011.

52. Qin ZF, Zhang HY, Feng G, Christie P, Zhang JL, Li XL, et al. Soil phosphorus availability modifies the relationship between AM fungal diversity and mycorrhizal benefits to maize in an agricultural soil. Soil Biology and Biochemistry. 2020;144:9. doi: 10.1016/j.soilbio.2020.107790. PMID: WOS:000526888500018.

53. Tao R. Impacts of organic fertilization with a drip irrigation system on bacterial and fungal communities in cotton field. Agricultural systems. 2020;v. 182:2020 v.182. doi: 10.1016/j.agsy.2020.102820. PMID: 6868843.

54. Xu PD, Liu YR, Zhu J, Shi L, Fu QL, Chen JZ, et al. Influence mechanisms of long-term fertilizations on the mineralization of organic matter in Ultisol. Soil and Tillage Research. 2020;201:8. doi: 10.1016/j.still.2020.104594. PMID: WOS:000531094800002.

55. Zhu Z, Bai Y, Lv M, Tian G, Zhang X, Li L, et al. Soil Fertility, Microbial Biomass, and Microbial Functional Diversity Responses to Four Years Fertilization in an Apple Orchard in North China. Horticultural Plant Journal. 2020. doi: <https://doi.org/10.1016/j.hpj.2020.06.003>.
